# Integrative analysis identifies TEAD4 as a universal prognostic biomarker in human cancers

**DOI:** 10.3389/fimmu.2025.1688563

**Published:** 2025-09-30

**Authors:** Mohan Liu, Yang Song, Yue Kang, Nan Xue, Jiuhan Zhao, Yi Jin, Chang Liu, Biao Wang

**Affiliations:** ^1^ Department of Biochemistry and Molecular Biology, School of Life Sciences, China Medical University, Shenyang, China; ^2^ Geriatrics Center, Fourth People’s Hospital of Shenyang, Shenyang, China; ^3^ Department of Breast Surgery, Cancer Hospital of Dalian University of Technology, Cancer Hospital of China Medical University, Liaoning Cancer Hospital & Institute, Shenyang, China; ^4^ Department of Orthodontics, School and Hospital of Stomatology of China Medical University, Liaoning Provincial Key Laboratory of Oral Disease, Shenyang, China; ^5^ Department of Neurology, The First Affiliated Hospital of China Medical University, Shenyang, Liaoning, China; ^6^ Department of Radiation Oncology, The First Affiliated Hospital of China Medical University, Shenyang, China

**Keywords:** TEAD4, pan-cancer analysis, prognostic marker, cancer stemness, drug resistance tumor immune microenvironment

## Abstract

**Introduction:**

TEA domain transcription factor 4 (TEAD4), a key effector of the Hippo signaling pathway, has been increasingly associated with tumorigenesis and cancer progression. Despite its recognized role, comprehensive pan-cancer analyses of TEAD4 expression patterns, prognostic significance, and therapeutic implications remain scarce.

**Methods:**

We conducted a systematic evaluation of TEAD4 across diverse tumor types using publicly available datasets, including TCGA. Analyses included gene expression profiling, prognostic correlation, functional enrichment, and drug sensitivity assessments. Additionally, in vitro assays were performed to validate the functional roles of TEAD4 in cancer cell behavior.

**Results:**

TEAD4 was significantly overexpressed in multiple cancers and associated with unfavorable prognosis. Functional enrichment analyses implicated TEAD4 in oncogenic processes such as proliferation, metastasis, stemness maintenance, and immune regulation. In vitro experiments confirmed that TEAD4 promotes cancer cell proliferation, migration, and stem cell-like properties, while TEAD4 knockdown reversed these phenotypes. TEAD4 expression correlated with genomic instability, epigenetic alterations, and remodeling of the tumor microenvironment. Drug sensitivity analysis indicated that elevated TEAD4 levels were linked to resistance against several chemotherapeutic agents. Furthermore, a prognostic model based on TEAD4 target gene expression successfully stratified patients by survival risk.

**Discussion:**

Our findings highlight the multifaceted roles of TEAD4 in cancer biology, emphasizing its contribution to tumor progression, therapy resistance, and patient outcomes. The evidence supports TEAD4 as a promising prognostic biomarker and therapeutic target, offering new avenues for translational cancer research.

## Highlights

- A comprehensive pan-cancer analysis reveals TEAD4 as a universal prognostic biomarker and therapeutic target.
- TEAD4 promotes tumor proliferation, migration, stemness, and therapeutic resistance *in vitro*.
- Multi-omics integration identifies TEAD4 as a key regulator of immune microenvironment remodeling.
- A novel TEAD4-based gene signature predicts patient survival across diverse cancer types.
- Findings suggest TEAD4 as a potential pan-cancer therapeutic target for precision oncology.

## 1 Introduction

Cancer remains one of the leading causes of morbidity and mortality worldwide, with therapeutic resistance and recurrence posing significant clinical challenges (1, 2). It’s known that cancer is a highly heterogeneous and multifactorial disease in which a series of complex genomic alterations cause the uncontrolled growth and proliferation, resulting in exceptional adaptability and resistance to therapy (3, 4). The high frequency of recurrences and the limited effectiveness of treatment options underscore the urgency of the need to uncover novel molecular mechanisms underlying tumor progression and therapeutic resistance. In this context, the identification of robust prognostic biomarkers has emerged as a crucial research priority. These biomarkers not only enhance the accuracy of clinical outcome prediction but also serve as critical entry points for developing individualized therapeutic strategies (5).

Transcription factors (TFs), acting as master regulators of gene expression, are central players in orchestrating tumor-related processes such as proliferation, metastasis, and therapy resistance (6). Among them, the TEA domain transcription factor (TEAD) family, particularly TEAD4, has garnered increasing attention due to its pervasive upregulation and strong association with unfavorable clinical outcomes across multiple cancers (7). Structurally, TEAD4 contains a TEA DNA-binding domain and a YAP-binding domain (YBD). The YBD includes three co-activator interaction interfaces, with Interface 3 (high-affinity, YAP-specific) and Interface 2 (moderate-affinity, shared by YAP, TAZ, VGLL1, and VGLL4) playing especially vital roles in mediating transcriptional activation (8).

Recent studies have implicated the TEAD4 transcription factor as a key driver of tumorigenesis in multiple cancer types (9–13, 14, 14), with its aberrant strongly correlated with aggressive disease and poor patient outcomes. It induces epithelial - mesenchymal transition (EMT), sustains cancer stem cell (CSC) traits, and remodels the tumor microenvironment (TME) by enhancing paracrine signaling, pseudopodia formation, and lymphangiogenesis (15). Additionally, TEAD4 reprograms cellular metabolism to favor tumor cell proliferation and metastatic dissemination (15). These findings implicate TEAD4 as a promising candidate for both biomarker development and targeted therapy across cancer types.

With the advancement of high-throughput sequencing and computational biology, pan-cancer analysis has become an indispensable strategy to systematically characterize gene expression and function across multiple tumor types (16). By integrating transcriptomic, epigenomic, genomic, and spatial single-cell data, pan-cancer approaches facilitate the identification of shared oncogenic features and tumor-specific molecular signatures (17). At the gene level, statistical tests (e.g., t-test, Wilcoxon, Kruskal-Wallis) and Pearson correlation analyses are employed to determine expression differences and inter-gene relationships. At the cellular level, algorithms such as ESTIMATE, CIBERSORT, EPIC, TIMER, and XCell quantify immune cell infiltration. At the phenotypic level, survival analysis and machine learning can be used to predict outcomes and identify biomarkers. Survival models like Kaplan-Meier and Cox regression can be used to estimate the time to an event, while machine learning models can learn complex relationships between features and outcomes.

This study presents a comprehensive pan-cancer analysis of TEAD4 by integrating data from the (epi)genome, transcriptome, and DNA methylome. It systematically investigates TEAD4’s expression patterns, mutational landscape, and functional roles across multiple tumor types. The analysis sheds light on the underlying regulatory mechanisms, including genomic alterations, remodeling of the immune microenvironment, and resistance to therapy. Furthermore, a predictive model based on TEAD4-associated genes is developed to support clinical risk stratification. These findings offer new perspectives on the oncogenic functions of TEAD4 and underscore its potential as a widespread prognostic biomarker and therapeutic target in cancer.

## 2 Materials and methods

### 2.1 Data acquisition and preprocessing

#### 2.1.1 Retrieval and harmonization of multi-omics data from public datasets

Multi-omics data pertaining to 33 distinct human cancer types were comprehensively interrogated, primarily sourced from The Cancer Genome Atlas (TCGA) program. The specific cancer cohorts included in this study, along with their respective TCGA abbreviations and sample sizes (n), were: acute myeloid leukemia (LAML, n=151), adrenocortical carcinoma (ACC, n=79), cholangiocarcinoma (CHOL, n=44), bladder cancer (BLCA, n=428), breast invasive carcinoma (BRCA, n=1226), cervical squamous cell carcinoma and endocervical adenocarcinoma (CESC, n=309), colon adenocarcinoma (COAD, n=514), uterine corpus endometrial carcinoma (UCEC, n=585), esophageal carcinoma (ESCA, n=198), glioblastoma multiforme (GBM, n=175), head and neck squamous cell carcinoma (HNSC, n=566), kidney chromophobe (KICH, n=91), kidney renal clear cell carcinoma (KIRC, n=610), kidney renal papillary cell carcinoma (KIRP, n=323), diffuse large B-cell lymphoma (DLBC, n=48), liver hepatocellular carcinoma (LIHC, n=424), brain lower grade glioma (LGG, n=534), lung sadenocarcinoma (LUAD, n=589), lung squamous cell carcinoma (LUSC, n=552), skin cutaneous melanoma (SKCM, n=473), mesothelioma (MESO, n=87), uveal melanoma (UVM, n=80), ovarian serous cystadenocarcinoma (OV, n=429), pancreatic adenocarcinoma (PAAD, n=183), pheochromocytoma and paraganglioma (PCPG, n=187), prostate adenocarcinoma (PRAD, n=554), rectum adenocarcinoma (READ, n=177), sarcoma (SARC, n=265), stomach adenocarcinoma (STAD, n=448), testicular germ cell tumors (TGCT, n=156), thymoma (THYM, n=122), thyroid carcinoma (THCA, n=572), and uterine carcinosarcoma (UCS, n=57).

Comprehensive multi-omics profiles, including transcriptomic data (RNA sequencing, FPKM values), epigenetic profiles (Illumina Infinium HumanMethylation450 BeadChip), copy number variation (CNV) segments (derived from Affymetrix Genome-Wide Human SNP Array 6.0 data using the ASCAT3 algorithm), and associated clinical phenotype information, were uniformly processed and downloaded via the UCSC Xena platform (https://xena.ucsc.edu/) (18). Gene identifiers were systematically converted to official HUGO Gene Nomenclature Committee (HGNC) gene symbols utilizing the R package org.Hs.eg.db (version 3.17.0). Subsequently, TEAD4 expression values were extracted from all processed samples for downstream analyses.

#### 2.1.2 Collection of single-cell RNA sequencing data

A total of 47 publicly available scRNA-seq datasets, collectively encompassing 30 distinct cancer types, were systematically retrieved from the Gene Expression Omnibus (GEO) and the European Molecular Biology Laboratory-European Bioinformatics Institute (EMBL-EBI) ArrayExpress. These datasets, detailed in **Supplementary Table 1**, were employed for high-resolution characterization of TEAD4 transcriptional signatures at the single-cell level. The sample data were normalized using the LogNormalize method. Subsequently, Harmony was applied to correct for batch effects, enabling robust comparative analysis across diverse samples.

#### 2.1.3 Collection of protein data

At the protein level, we evaluated the expression differences of TEAD4 protein between cancerous and normal tissues using the UALCAN database (http://ualcan.path.uab.edu/). Simultaneously, immunohistochemistry data for TEAD4 was obtained from the HPA database (https://www.proteinatlas.org/), and a standardized immunohistochemical scoring system was employed to quantitatively compare its expression levels in cancerous and normal tissues.

In our IHC scoring system, we adopted a three-dimensional evaluation criteria (1): antibody staining extent, categorized as high, medium, or low, assigned 3, 2, and 1 point(s) respectively (2);staining intensity, classified as positive, moderate, or negative, corresponding to 3, 2, and 1 point(s) respectively (3);the percentage of positive cells, with above 75% scoring 3 points, 25%–75% scoring 2 points, and below 25% scoring 1 point. The final IHC score is the product of the points from these three dimensions. Using tissue sections of normal and tumor tissues provided in the HPA database, we scored TEAD4 staining respectively and assessed the statistical difference. The distribution of IHC scores between groups was compared using the Mann-Whitney U test. P-values were adjusted for multiple comparisons using the Benjamini-Hochberg false discovery rate (FDR) correction.

It should be noted that the labels ‘High/Medium/Low/Negative’ shown in the lower right corner of the images are based on the original classification of antibody staining extent by the HPA database, and not our custom-defined categories.

#### 2.1.4 Acquisition of spatial transcriptomic data

Spatial transcriptomic profiles for CRC and PRAD were obtained from datasets generated using the 10x Genomics Visium platform. Additionally, spatial transcriptomic datasets for other malignancies, including BRCA, UCEC, PAAD, and GBM, were retrieved from GEO under accession numbers GSE243275, GSE225690, GSE194329, and GSE235315, respectively. These datasets are listed in **Supplementary Table 2**. This integrated spatial omics resource facilitated the systematic evaluation of TEAD4 expression patterns in relation to the spatial distribution of immune cells within TME.

### 2.2 Differential expression analysis of TEAD4

To investigate the pleiotropic disease associations of TEAD4, the Open Targets Platform (https://platform.opentargets.org/) was initially employed. Genotype-phenotype correlations were visualized using multivariate bubble plots, with a statistical significance threshold of *P* < 0.05 (19).

The ‘gene_DE’ module of TIMER 2.0 (Tumor Immune Estimation Resource, version 2.0; http://timer.cistrome.org/) was utilized to systematically evaluate differences in TEAD4 expression between tumor tissues and matched adjacent normal samples across the TCGA pan-cancer cohort (20). For cancer types lacking a sufficient number of matched adjacent normal samples within TCGA, complementary differential expression analyses were performed using the ‘Expression Analysis - Box Plots’ module of GEPIA 2.0 (Gene Expression Profiling Interactive Analysis, version 2.0; http://gepia2.cancer-pku.cn/). Stringent statistical thresholds of *P* < 0.01 and an absolute log_2_(Fold Change) > 1 were applied for these analyses (21). TEAD4 expression was also analyzed across different clinical stages and pathological grades within each cancer type.

### 2.3 Association of TEAD4 expression with clinical outcomes

Prognostic analyses were conducted using the survival R package (version 3.5-5). Patients were stratified into high- and low-TEAD4 expression groups based on the median TEAD4 mRNA expression level within each cancer type. Survival times were standardized to years (days/365) for clinical relevance. Univariate Cox proportional hazards regression analysis, implemented via the coxph() function, was employed to quantify the association between TEAD4 expression and patient outcomes, including Overall Survival (OS), Disease-Specific Survival (DSS), and Progression-Free Interval (PFI). Hazard Ratios (HR) with corresponding 95% Confidence Intervals (CI) were calculated. To visualize survival differences, Kaplan-Meier curves were generated using the survfit() function, and the statistical significance of differences between high- and low-expression groups was assessed using the log-rank test.

### 2.4 Landscape of TEAD4 genomic alterations and prognostic impact

The genomic alteration profile of TEAD4 across various cancers was investigated using the cBioPortal for Cancer Genomics platform (https://www.cbioportal.org/) (22). Within the ‘Quick select’ interface, the ‘TCGA Pan-Cancer Atlas’ study was accessed, and the ‘TEAD4’ gene was queried to examine its alteration characteristics. The ‘Cancer Types Summary’ module was utilized to systematically evaluate the frequency of major alteration types, including somatic mutations, amplifications, and deep deletions.

For methylation analysis, probes located within the TEAD4 promoter region, identified based on its genomic coordinates (Chr12: 2,959,330-3,040,676, GRCh38/hg38), were extracted from the TCGA Infinium HumanMethylation450K dataset. The methylation level of the TEAD4 promoter in each sample was represented by the average β-value of these probes. Samples were subsequently stratified into high- and low-methylation groups based on the median β-value, followed by Kaplan-Meier survival analysis to assess the association between TEAD4 promoter methylation status and patient prognosis.

For CNV evaluation, copy number data corresponding to the TEAD4 genomic region were extracted from TCGA. The average CNV value across this region was calculated for each sample to determine the extent of copy number alteration. Utilizing median CNV values as the stratification cutoff, samples were classified into high- and low-CNV groups. Kaplan-Meier survival analysis was then performed to investigate the correlation between TEAD4 copy number variation and clinical outcomes.

### 2.5 Curation of TEAD4 target genes

Putative TEAD4 target genes were systematically curated through the integration of information from three authoritative databases: the Encyclopedia of DNA Elements (ENCODE) project (https://www.encodeproject.org), ChEA3 (ChIP-X Enrichment Analysis version 3; https://maayanlab.cloud/chea3), and hTFtarget (Human Transcription Factor Target; http://bioinfo.life.hust.edu.cn/hTFtarget) (23–25). This integrative approach was designed to ensure comprehensive coverage of both experimentally validated and computationally predicted TEAD4-regulated target genes.

### 2.6 Functional enrichment analysis of TEAD4 target genes

TEAD4 target genes were functionally characterized via Gene Ontology (GO) annotation (Biological Process, Cellular Component, and Molecular Function) and Kyoto Encyclopedia of Genes and Genomes (KEGG) pathway analysis, utilizing the DAVID (Database for Annotation, Visualization and Integrated Discovery) platform (version 2021) (26). Statistically significant enrichment results were visualized using the ggplot2 R package (version 3.5.0) to illustrate key biological processes and signaling pathways.

Gene Set Enrichment Analysis (GSEA) was performed using the clusterProfiler R package (version 4.8.3) (27). Tumor samples were stratified into high-TEAD4 and low-TEAD4 expression groups based on median expression values. Genes were ranked according to their differential expression between these two groups. Enrichment analysis was subsequently conducted against reference gene sets from the Molecular Signatures Database (MSigDB, version 7.4 and v2024.1.Hs), including h.all.v7.4.symbols.gmt (Hallmark gene sets), c2.all.v2024.1.Hs.symbols.gmt (Curated gene sets, encompassing KEGG, Reactome, BioCarta), and c5.all.v2024.1.Hs.symbols.gmt (GO gene sets). Results with a *P*-value < 0.05 were considered statistically significant and were visualized using bubble plots and ridge plots.

### 2.7 Assessment of TEAD4 in relation to genomic stability

#### 2.7.1 Impact of TEAD4 expression on driver gene mutation rates

The ‘Mutational Landscape’ module of the CAMOIP (Cancer Multi-omics Atlas and Online Interactive Platform) database (http://www.camoip.net/) was employed to systematically compare tumor driver gene mutation rates between high-TEAD4 and low-TEAD4 expression groups across various cancer types (28).

#### 2.7.2 Correlation analyses of TEAD4 with cancer-associated genomic alterations

The maftools R package was utilized to extract and quantify four key genomic instability characteristics from TCGA somatic mutation data: tumor mutation burden (TMB), microsatellite instability (MSI) status (where available), homologous recombination deficiency (HRD) scores, and ploidy estimates. Pearson correlation coefficients were calculated to assess the potential associations between TEAD4 mRNA expression levels and each of these genomic features.

### 2.8 Investigation of TEAD4’s influence on the tumor immune microenvironment

#### 2.8.1 Preliminary analysis of TEAD4’s impact on tumor immunity

The association of TEAD4 expression with the six established immune subtypes (C1-C6, as defined by Thorsson et al.) was analyzed, and its expression levels were compared across these subtypes in a pan-cancer manner using the Subtype module of TISIDB (an integrated repository portal for Tumor-Immune System Interactions; http://cis.hku.hk/TISIDB/) (29).

Subsequently, co-expression relationships between TEAD4 and a panel of key immune regulators, including immune checkpoint molecules, chemokines, chemokine receptors, immune stimulators, and immune inhibitors, were systematically characterized. For gene expression arrays with multiple probes mapping to the same gene, technical replicates were consolidated by averaging expression values using the avereps() function from the limma R package to generate unique expression profiles per sample. Pairwise Pearson correlation coefficients were then computed using the cor.test() function in R to quantify TEAD4-immunogene associations, with statistical significance assessed at *P* < 0.05.

#### 2.8.2 Effect of TEAD4 on stromal and immune cell infiltration

The role of TEAD4 in modulating pan-cancer TME infiltration was initially investigated by calculating ESTIMATE (Estimation of STromal and Immune cells in MAlignant Tumours using Expression data) scores. Stromal and immune scores, along with an estimate of tumor purity, were calculated for each TCGA sample using the estimate R package.

Subsequently, correlations between TEAD4 expression and the infiltration levels of various immune cell types were assessed using the CIBERSORT algorithm (https://cibersort.stanford.edu/) with the LM22 signature matrix and 1000 permutations. TIMER 2.0 was further employed to explore the intercorrelation between TEAD4 expression and the infiltration of specific immune cell populations, such as macrophages, regulatory T cells (Tregs), and CD8+ T cells, utilizing five different deconvolution algorithms (TIMER, CIBERSORT, CIBERSORT-ABS, EPIC, quanTIseq, xCell).

#### 2.8.3 Single-cell transcriptomic profiling of TEAD4 across diverse cancers

Through an integrated analysis of 47 independent scRNA-seq datasets spanning 30 cancer types, TEAD4 transcriptional dynamics were systematically characterized at single-cell resolution using the Seurat R package (version 5.0.1). This pan-cancer deconvolution aimed to reveal cell-type-specific TEAD4 activity across functionally annotated cell populations within the TME, including malignant epithelial cells along the EMT spectrum, various immune cell subsets (e.g., T/NK cell exhaustion programs, B cell maturation stages, macrophage polarization states), and stromal compartments (cancer-associated fibroblast (CAF) subtypes, angiogenic endothelial clusters).

#### 2.8.4 Spatial transcriptomic profiling of TEAD4 in tumor microenvironments

Acquired spatial transcriptomic datasets were analyzed using Seurat (version 5.0.1) to delineate the spatial expression architecture of TEAD4 within intact tumor tissues. The analytical pipeline specifically focused on characterizing TEAD4’s spatial co-localization patterns with distinct immune cell populations, particularly macrophage polarization states (M0/M1/M2). This was achieved through neighborhood enrichment analysis and ligand-receptor interaction mapping. Data processing included spot deconvolution using SPOTlight (version 0.1.6) to resolve cellular compositions within each spatial spot, followed by spatial autocorrelation analysis to identify significant TEAD4 expression hotspots and their association with immune cell niches.

### 2.9 Assessment of TEAD4’s impact on cancer therapy sensitivity

#### 2.9.1 Correlation of TEAD4 with DNA damage repair pathways as a proxy for radiotherapy sensitivity

To infer potential associations with radiotherapy sensitivity, gene sets related to homologous recombination repair (HRR; 34 genes) and mismatch repair (MMR; 22 genes) were retrieved. Pearson correlation coefficients were calculated between TEAD4 expression and the expression of each gene within these DNA repair pathways across TCGA cancer types.

#### 2.9.2 Effect of TEAD4 expression on chemotherapy drug sensitivity

The CellMiner™ database (https://discover.nci.nih.gov/cellminer/) was utilized to explore the relationship between TEAD4 expression levels (NCI-60 cancer cell line panel) and sensitivity to a wide range of FDA-approved chemotherapy drugs and other pharmacological compounds (30). Drug sensitivity was typically measured as GI_50_ (concentration inhibiting growth by 50%). Differences in drug sensitivity between TEAD4-high and TEAD4-low expressing cell lines were assessed.

The Genomics of Drug Sensitivity in Cancer (GDSC) (https://www.cancerrxgene.org/) is currently the largest public database on drug sensitivity in cancer cell lines. To investigate the relationship between gene expression and drug sensitivity, it is necessary to obtain gene expression profiles across different cell lines, as well as IC50 or AUC values of various drugs for each cell line. This study uses the GDSC2 database as the training set, in which GDSC2_exp is a standardized expression matrix comprising 17,419 genes and 805 cell lines, while GDSC_drug provides IC50 values of 198 drugs across the 805 cell lines. A predictive model was built based on this training set and then applied to expression data from BLCA, KIRC, LUAD, and PAAD samples in the GDC TCGA, which were downloaded from the UCSC Xena website. Finally, the difference in IC50 values between high and low TEAD4 expression groups was compared, and its statistical significance was evaluated.

### 2.10 Construction and validation of a patient prognostic prediction model based on TEAD4 target genes

This study focused on patient samples from cancer types wherein TEAD4 expression demonstrated a significant impact on patient survival in our initial analyses. For each selected cancer type, Pearson correlation analysis was conducted between TEAD4 expression and its curated potential target genes. Key TEAD4-correlated target genes were defined as those exhibiting an absolute correlation coefficient > 0.1 with TEAD4 expression and were subsequently selected for prognostic model construction.

In the data preprocessing phase, samples from each selected cancer type were randomly divided into a training set (70% of samples) and a test set (30% of samples). Within the training set, univariate Cox regression analysis was performed to identify prognostically significant TEAD4 target genes. Subsequently, a multivariate Cox proportional hazards model was constructed using a stepwise backward elimination approach based on the Akaike Information Criterion (AIC) to select the optimal combination of genes for the prognostic signature. The risk score for each patient was calculated as a linear combination of the expression levels of the selected genes, weighted by their respective Cox regression coefficients. Patients were then stratified into high-risk and low-risk groups based on the median risk score. The prognostic performance of the model was evaluated in both the training and test sets using Kaplan-Meier survival analysis (log-rank test) and time-dependent Receiver Operating Characteristic (ROC) curve analysis (AUC values for 1-, 3-, and 5-year survival).

### 2.11 Cell culture and reagents

Human cancer cell lines A549 (lung adenocarcinoma), Panc-1 (pancreatic ductal adenocarcinoma), 769-P (renal cell carcinoma), and the human embryonic kidney cell line HEK293T were obtained from the American Type Culture Collection (ATCC, Manassas, VA, USA). A549, Panc-1, and HEK293T cells were cultured in Dulbecco’s Modified Eagle’s Medium (DMEM; Gibco, Grand Island, NY, USA) supplemented with 10% fetal bovine serum (FBS; Gibco) and 1% penicillin-streptomycin (Gibco). 769-P cells were maintained in RPMI-1640 medium (Gibco) supplemented with 10% FBS and 1% penicillin-streptomycin. All cell lines were cultured in a humidified incubator at 37°C with 5% CO_2_.

### 2.12 Establishment of TEAD4 overexpression and knockdown cell lines

For TEAD4 overexpression, the full-length human TEAD4 cDNA was cloned into the pLVX-Puro lentiviral vector (Clontech, Mountain View, CA, USA). For TEAD4 knockdown, short hairpin RNAs (shRNAs) targeting human TEAD4 (shTEAD4) and a non-targeting control shRNA (shCtrl) were cloned into the pLKO.1-Puro lentiviral vector (Addgene, Watertown, MA, USA). Lentiviral particles were produced in HEK293T cells by co-transfecting the respective lentiviral plasmids with packaging plasmids (psPAX2 and pMD2.G; Addgene) using Lipofectamine 3000 (Invitrogen, Carlsbad, CA, USA) according to the manufacturer’s instructions. Supernatants containing viral particles were harvested 48 and 72 hours post-transfection, filtered, and used to infect target cells in the presence of polybrene (8 µg/mL; Sigma-Aldrich, St. Louis, MO, USA). Stable cell lines were selected using puromycin (2 µg/mL; Sigma-Aldrich) for at least 7 days. The efficiency of TEAD4 overexpression and knockdown was confirmed by Western blot analysis.

### 2.13 Western blot analysis

Cells were lysed in RIPA buffer (Beyotime, Shanghai, China) supplemented with protease and phosphatase inhibitor cocktails (Roche, Basel, Switzerland). Protein concentrations were determined using the BCA Protein Assay Kit (Beyotime). Equal amounts of protein (20-30 µg) were separated by SDS-PAGE and transferred to polyvinylidene difluoride (PVDF) membranes (Millipore, Billerica, MA, USA). Membranes were blocked with 5% non-fat milk in Tris-buffered saline with 0.1% Tween 20 (TBST) for 1 hour at room temperature and then incubated overnight at 4°C with primary antibodies against TEAD4 (1:1000; Abcam, Cambridge, UK, ab124957) and GAPDH (1:5000; Proteintech, Rosemont, IL, USA, 60004-1-Ig). After washing with TBST, membranes were incubated with HRP-conjugated secondary antibodies (1:5000; Proteintech) for 1 hour at room temperature. Protein bands were visualized using an ECL Western Blotting Substrate (Bio-Rad, Hercules, CA, USA) and imaged with a ChemiDoc XRS+ System (Bio-Rad).

### 2.14 RNA extraction and quantitative real-time PCR

Total RNA was extracted from cells using TRIzol reagent (Invitrogen) according to the manufacturer’s protocol. cDNA was synthesized from 1 µg of total RNA using the PrimeScript™ RT Reagent Kit with gDNA Eraser (Takara Bio, Shiga, Japan). qRT-PCR was performed using TB Green^®^ Premix Ex Taq™ II (Tli RNaseH Plus) (Takara Bio) on a QuantStudio 5 Real-Time PCR System (Applied Biosystems, Foster City, CA, USA). The relative expression of target genes was normalized to GAPDH expression using the 2^-ΔΔCt^ method. Primer sequences used for qRT-PCR are listed in **Supplementary Table 3**.

### 2.15 Cell proliferation assay

Cell proliferation was assessed using the Cell Counting Kit-8 (CCK-8; Dojindo Molecular Technologies, Kumamoto, Japan). Cells (2 × 10³ cells/well) were seeded into 96-well plates. At indicated time points (0, 24, 48, 72, and 96 hours), 10 µL of CCK-8 solution was added to each well, and plates were incubated for 2 hours at 37°C. The absorbance at 450 nm was measured using a microplate reader (BioTek Instruments, Winooski, VT, USA). Each experiment was performed in triplicate.

### 2.16 Wound healing assay

Cells were seeded into 6-well plates and grown to confluence. A sterile 200 µL pipette tip was used to create a linear scratch (wound) in the cell monolayer. Detached cells were removed by washing with PBS. Cells were then cultured in serum-free medium. Images of the wound area were captured at 0 and 24 hours (or other indicated time points) using an inverted microscope (Olympus, Tokyo, Japan). The wound closure rate was quantified using ImageJ software (NIH, Bethesda, MD, USA) and calculated as: (Initial wound area - Wound area at time X)/Initial wound area × 100%.

### 2.17 Colony formation

The cancer cell line was digested with 0.25% trypsin and resuspended into a single-cell suspension for cell counting. Then, 500 cells were seeded into a well of 6-well plate and cultured in a constant-temperature incubator for 1–2 weeks. After individual cell clones had formed, the original culture medium was discarded, and the cells were washed 2–3 times with PBS. Next, 2 ml of 4% paraformaldehyde was added to fix the cells for 20 minutes. After another 2–3 washes with PBS, 2 ml of 1% crystal violet was added to stain the cells for 20 minutes. The stain was then rinsed off with water, and the cells were air-dried at room temperature before being photographed for counting. The number of clones was quantified using ImageJ software, and statistical graphs were generated with GraphPad.

### 2.18 Statistical analysis

All statistical analyses were performed using R software (version 4.2.1 or later; R Foundation for Statistical Computing, Vienna, Austria) and GraphPad Prism (version 9.0; GraphPad Software, San Diego, CA, USA). Data are presented as mean ± standard deviation (SD) from at least three independent experiments, unless otherwise specified. Differences between two groups were analyzed using Student’s t-test or Wilcoxon rank-sum test, as appropriate. Comparisons among multiple groups were performed using one-way ANOVA followed by Tukey’s *post hoc* test or Kruskal-Wallis test followed by Dunn’s *post hoc* test. Correlation analyses were performed using Pearson or Spearman correlation coefficients. Survival analyses were conducted using Kaplan-Meier curves with the log-rank test and Cox proportional hazards regression models. A two-sided *P*-value < 0.05 was considered statistically significant for all analyses. Specific statistical methods are detailed within the relevant sections or figure legends.

## 3 Results

### 3.1 TEAD4 functional profiling and expression analysis

**Figure 1:**
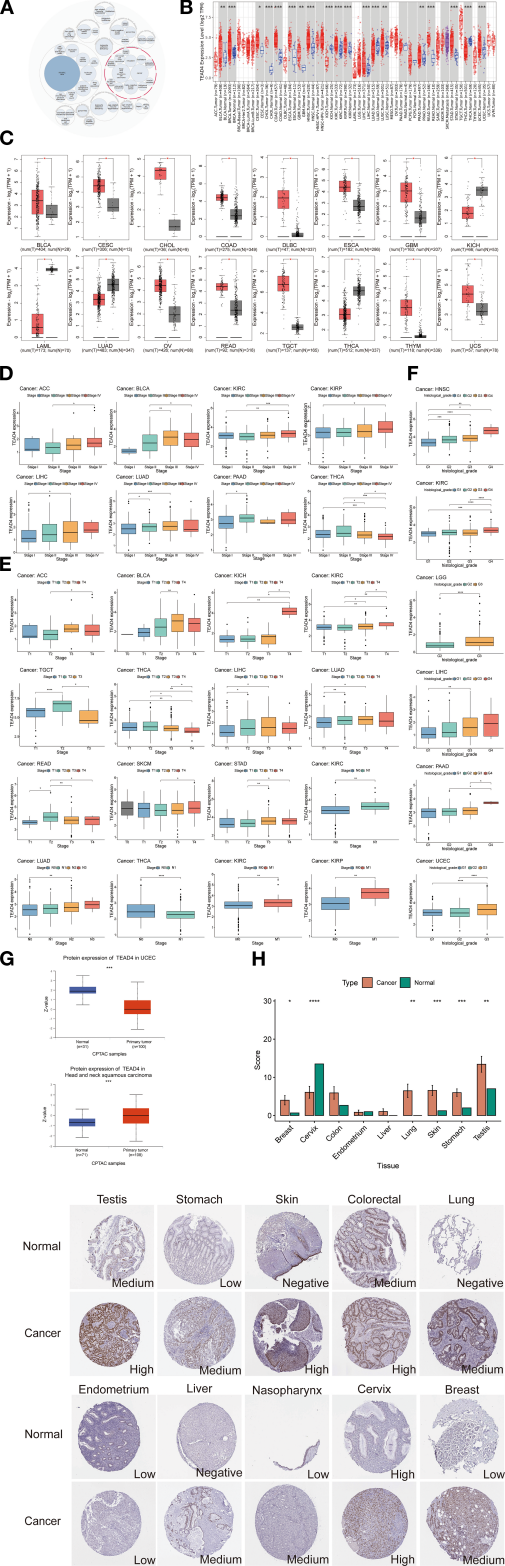
TEAD4 functional profiling and expression analysis. **(A)** Open Targets platform revealed significant associations between TEAD4 and multiple diseases, where the circle sizes represent the strength of association scores. **(B)** TIMER 2.0 database revealed significant differential expression of TEAD4 between tumor tissues and adjacent normal tissues. Tumors and adjacent tissues are colored in red and blue, respectively. **(C)** Boxplots represent the differential expression of TEAD4 mRNA log2 expression levels between tumors and normal tissues in 16 cancer types via GEPIA2.0. **(D)** Differential TEAD4 expression across cancer stages. **(E)** Differential TEAD4 expression across cancer TNM stages. **(F)** Differential TEAD4 expression across tumor grades. **(G)** UALCAN database reveals differential TEAD4 protein expression between tumor and normal tissues. **(H)** HPA database documents TEAD4 protein distribution patterns and expression intensity (quantified by immunohistochemical staining scores) in malignant versus normal tissues (*P < 0.05, **P < 0.01, ***P < 0.001, ****P < 0.0001).

The Open Targets Platform, a comprehensive data integration tool that aggregates publicly available datasets was employed to identify and prioritize potential therapeutic drug targets. Our analysis of TEAD4-associated biological functions indicates its significant involvement in several cancer-related processes, notably in BRCA, NSCLC, CRC, and HNSC (**Figure 1A**). To further elucidate the role of TEAD4 in oncogenesis, we analyzed its expression profiles across multiple cancer types. The data revealed that TEAD4 mRNA expression was significantly upregulated in 14 cancer types compared to adjacent normal tissues. These included BLCA, BRCA, CESC, CHOL, COAD, ESCA, GBM, HNSC, LIHC, LUSC, PRAD, READ, STAD, and UCEC. In contrast, TEAD4 expression was significantly downregulated in five cancer types: KICH, KIRC, KIRP, LUAD, and THCA (P < 0.05) (**Figure 1B**).

To validate these findings, we utilized the GEPIA2.0 database, which confirmed significant TEAD4 mRNA upregulation in 12 cancer types relative to normal tissues: BLCA, CESC, CHOL, COAD, DLBC, ESCA, GBM, OV, READ, TGCT, THYM, and UCS. Conversely, four cancer types—KICH, LAML, LUAD, and THCA - showed significant downregulation (P < 0.05) (**Figure 1C**). The high degree of concordance between datasets supports the robustness and reliability of these results. Further analysis of TEAD4 expression across cancer stages and grades revealed its strong association with cancer progression. In stage-wise comparisons, TEAD4 mRNA levels were significantly elevated in advanced stages of ACC, BLCA, KICH, KIRC, LUAD, and STAD compared to early stages. Notably, THCA exhibited the opposite trend, with reduced expression in more advanced stages (P < 0.05) (**Figure 1D**).

In the context of TNM classification—where T denotes primary tumor size or extent, N indicates regional lymph node involvement, and M refers to distant metastasis—consistent expression patterns were observed. Specifically, higher TEAD4 mRNA expression was noted in advanced T stages of ACC, BLCA, KICH, KIRC, LIHC, LUAD, READ, SKCM, and STAD, while TGCT showed reduced expression in more advanced T stages. Elevated TEAD4 expression was also observed in advanced N stages of KIRC and LUAD, as well as in advanced M stages of KIRC and KIRP (P < 0.05) (**Figure 1E**). With regard to tumor grade—defined by cellular differentiation and histopathological heterogeneity—higher TEAD4 mRNA levels were significantly associated with high-grade tumors in UCEC, LIHC, LGG, and KIRC, compared to their lower-grade counterparts (P < 0.05) (**Figure 1F**). Protein-level validation is critical for bridging transcriptional data with clinical application. To assess TEAD4 protein expression, we analyzed data from the UALCAN and Human Protein Atlas (HPA) databases. According to UALCAN, TEAD4 protein levels were significantly elevated in HNSC but reduced in UCEC when compared to normal tissues (P < 0.05) (**Figure 1G**). Complementary immunohistochemical (IHC) data from the HPA further supported these findings, revealing increased TEAD4 protein expression in BRCA, LUAD, SKCM, STAD, and TGCT relative to normal controls (**Figure 1H**).

In conclusion, integrative analysis across multiple databases demonstrates that TEAD4 displays distinct expression profiles across various cancer types. Importantly, TEAD4 is predominantly upregulated in cancer, and its expression often correlates with advanced stages and poor differentiation, suggesting a potential role in tumor progression.

### 3.2 Prognostic impact of TEAD4 expression across pan-cancer types

**Figure 2:**
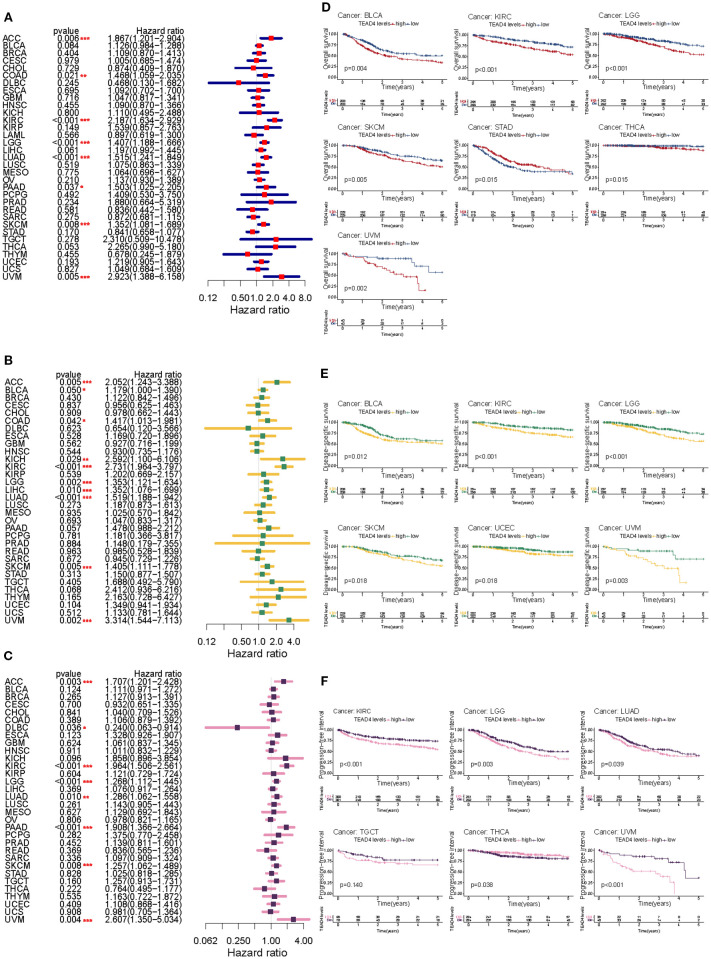
Prognostic impact of TEAD4 expression across pan-cancer types. **(A–C)** Forest plots demonstrate the HR relationships between TEAD4 expression levels and OS, DSS, and PFI in cancer patients. Point estimates with 95% confidence intervals quantify the prognostic impact strength of TEAD4 expression across various tumor types. **(D–F)** Kaplan-Meier survival curves compare prognostic outcomes between TEAD4-high and TEAD4-low expression groups across different survival endpoints (OS, DSS, PFI), revealing the significant influence of TEAD4 expression levels on long-term patient survival outcomes (*P < 0.05, **P < 0.01, ***P < 0.001, ****P < 0.0001).

We assessed the prognostic relevance of TEAD4 mRNA expression across multiple cancer types using univariate Cox regression analysis and Kaplan–Meier survival analysis. In the Cox regression model, we calculated hazard ratios (HRs) along with their 95% confidence intervals (CIs) and log-rank P values, and visualized the results using forest plots across various survival metrics. For OS, elevated TEAD4 mRNA expression was significantly associated with poorer prognosis in patients with ACC, BLCA, KIRC, LGG, LIHC, LUAD, PAAD, SKCM, and UVM (P < 0.05) (**Figure 2A**).

Similarly, high TEAD4 expression levels predicted worse disease-specific survival (DSS) in ACC, KIRC, LGG, LIHC, LUAD, SKCM, and UVM (P < 0.05) (**Figure 2B**).

For PFI, increased TEAD4 mRNA expression was a significant risk factor in ACC, KIRC, LGG, LUAD, PAAD, SKCM, and UVM (P < 0.05) (**Figure 2C**). We further validated these findings using Kaplan–Meier survival curves, which illustrated survival differences between patients with high versus low TEAD4 expression, highlighting the cancer-type-specific nature of its prognostic relevance.

For OS, elevated TEAD4 expression was significantly associated with reduced survival in BLCA, KIRC, LUAD, and UVM patients. Conversely, STAD patients with high TEAD4 expression unexpectedly exhibited improved survival outcomes (P < 0.05) (**Figure 2D**). In terms of DSS, high TEAD4 expression was linked to worse prognosis in BLCA, KIRC, LGG, READ, UCEC, and UVM (P < 0.05) (**Figure 2E**). Regarding PFI, elevated TEAD4 expression was significantly correlated with unfavorable outcomes in KIRC, LGG, LUAD, PAAD, TGCT, and UVM, while showing a favorable association in THCA (P < 0.05) (**Figure 2F**).

Collectively, these findings underscore the complex, cancer-specific prognostic role of TEAD4 mRNA expression across diverse malignancies and survival endpoints. This suggests TEAD4 may serve as both a potential prognostic biomarker and a therapeutic target in precision oncology.

### 3.3 Prognostic significance of TEAD4 expression across human cancers

To evaluate the prognostic value of TEAD4, univariate Cox proportional hazards regression analysis and Kaplan-Meier survival analysis were performed across the TCGA pan-cancer cohort, assessing Overall Survival (OS), DSS, and PFI. Elevated TEAD4 expression was significantly associated with poorer OS in 10 cancer types, including ACC, BLCA, BRCA, CESC, KIRC, KIRP, LGG, LIHC, LUAD, and MESO (*P* < 0.05) (**Figure 2A**).

Similarly, Kaplan-Meier survival curves further illustrated that patients with high TEAD4 expression exhibited significantly shorter OS compared to those with low TEAD4 expression in these malignancies (**Figure 2B**). Regarding DSS, high TEAD4 expression correlated with unfavorable outcomes in 9 cancer types: ACC, BLCA, BRCA, CESC, KIRC, KIRP, LGG, LIHC, and MESO (*P* < 0.05) (**Figure 2C**). Kaplan-Meier analysis confirmed these findings, showing reduced DSS in the TEAD4-high groups (**Figure 2D**). For PFI, increased TEAD4 expression was linked to worse prognosis in 10 cancer types: ACC, BLCA, BRCA, CESC, KIRC, KIRP, LGG, LIHC, PRAD, and UCEC (*P* < 0.05) (**Figure 2E**). Consistently, Kaplan-Meier curves demonstrated shorter PFI for patients with high TEAD4 expression in these cancers (**Figure 2F**).

These comprehensive survival analyses consistently demonstrate that elevated TEAD4 expression serves as an adverse prognostic indicator across a broad spectrum of human cancers, highlighting its potential clinical utility in risk stratification.

### 3.4 Genomic variation of TEAD4 and its impact on prognosis

**Figure 3:**
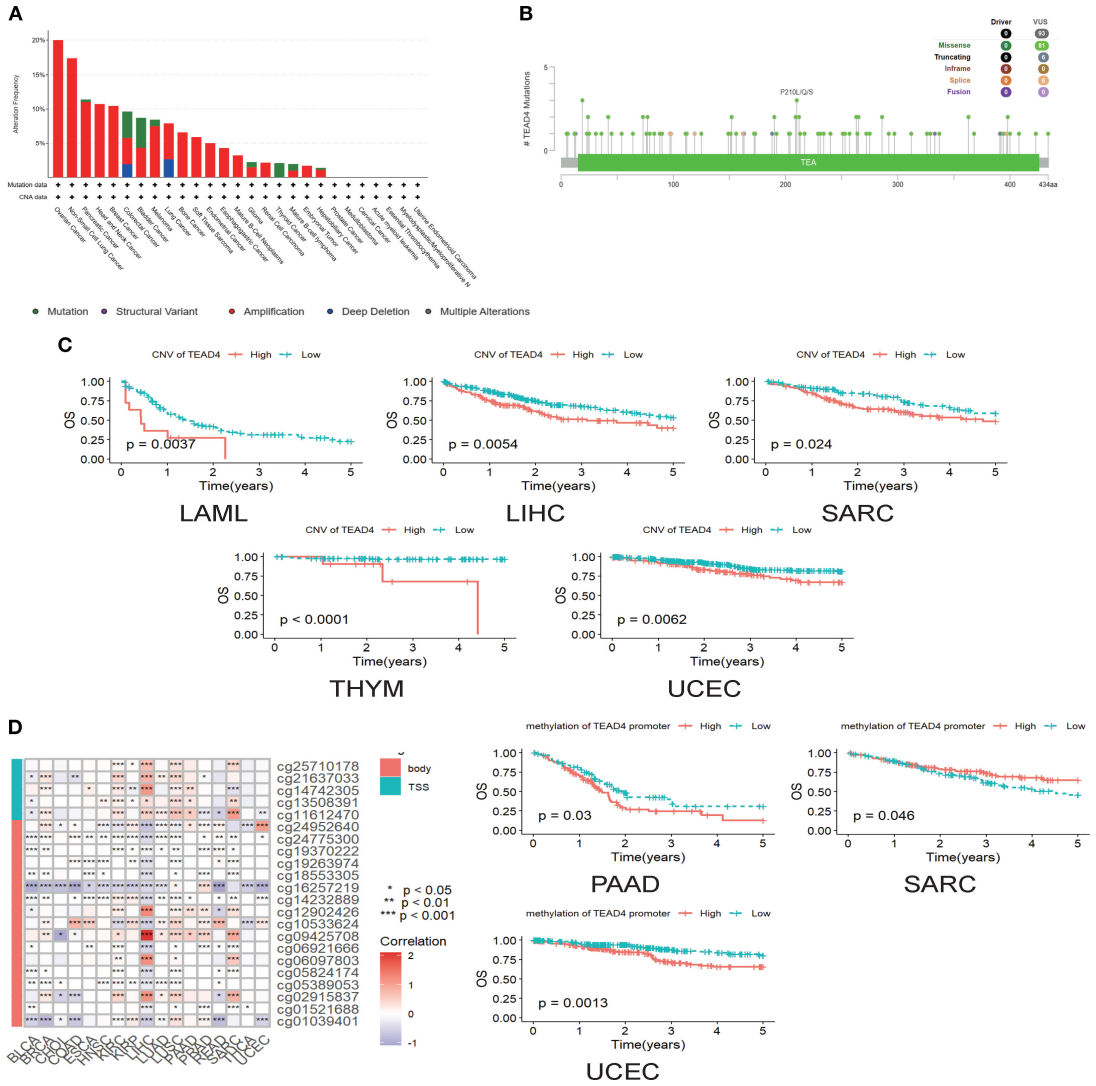
Genomic variation of TEAD4 and its impact on prognosis. **(A)** cBioPortal database illustrates the frequency distribution of TEAD4 genomic alterations across multiple cancer types, revealing tumor-specific genomic variation patterns. **(B)** Lollipop plot displays the panoramic distribution of TEAD4 single-nucleotide variations, visually depicting mutation sites and their frequencies. **(C)** Kaplan-Meier survival curves demonstrate the prognostic impact of TEAD4 CNVs on patient outcomes. **(D)** Heatmap presents methylation probes and their correlation with TEAD4 expression levels, while Kaplan-Meier curves further reveal the prognostic significance of TEAD4 promoter methylation levels (*P < 0.05, **P < 0.01, ***P < 0.001, ****P < 0.0001).

Comprehensive analysis of genomic alterations provided important mechanistic insights into the oncogenic role of TEAD4. Utilizing the cBioPortal database, we systematically characterized TEAD4 genomic alterations across a range of cancer types. Among these, gene amplification was the most prevalent alteration, particularly in ovarian serous cystadenocarcinoma, where the amplification frequency reached 20%, followed by non-small cell lung cancer (NSCLC) and pancreatic adenocarcinoma (PAAD) (**Figure 3A**).

Further analysis identified 93 single-nucleotide variants (SNVs) within the TEAD4 gene, all of which were classified as variants of uncertain significance. Of these, 81 were missense mutations, with the highest mutation frequency observed at position 210, where alanine was frequently substituted by leucine, glutamine, or serine (**Figure 3B**).

Clinically, copy number variations (CNVs) in TEAD4 exhibited significant associations with patient outcomes. Increased CNV levels were correlated with poorer prognosis in several cancers, including sarcoma (SARC), acute myeloid leukemia (LAML), liver hepatocellular carcinoma (LIHC), thymoma (THYM), and uterine corpus endometrial carcinoma (UCEC) (P < 0.05) (**Figure 3C**).

Interestingly, although TEAD4 promoter methylation generally showed a positive correlation with mRNA expression, its prognostic implications varied by cancer type. High methylation levels were associated with worse outcomes in PAAD and UCEC, whereas in SARC, higher methylation levels were linked to better prognosis (P < 0.05) (**Figure 3D**).

This integrative genomic-phenotypic analysis not only delineates the mutation and alteration landscape of TEAD4 across cancers but also reinforces its prognostic relevance, providing a mechanistic basis for the expression–prognosis correlations described in earlier sections.

### 3.5 Functional enrichment analysis of TEAD4

As a transcription factor, TEAD4 orchestrates a wide range of biological processes by regulating downstream target genes. To systematically characterize the functional network of TEAD4, we integrated its target genes from the ENCODE, ChEA3, and hTFtarget databases. These targets were then subjected to GO and KEGG pathway enrichment analyses, ranked according to the number of enriched genes.

**Figure 4:**
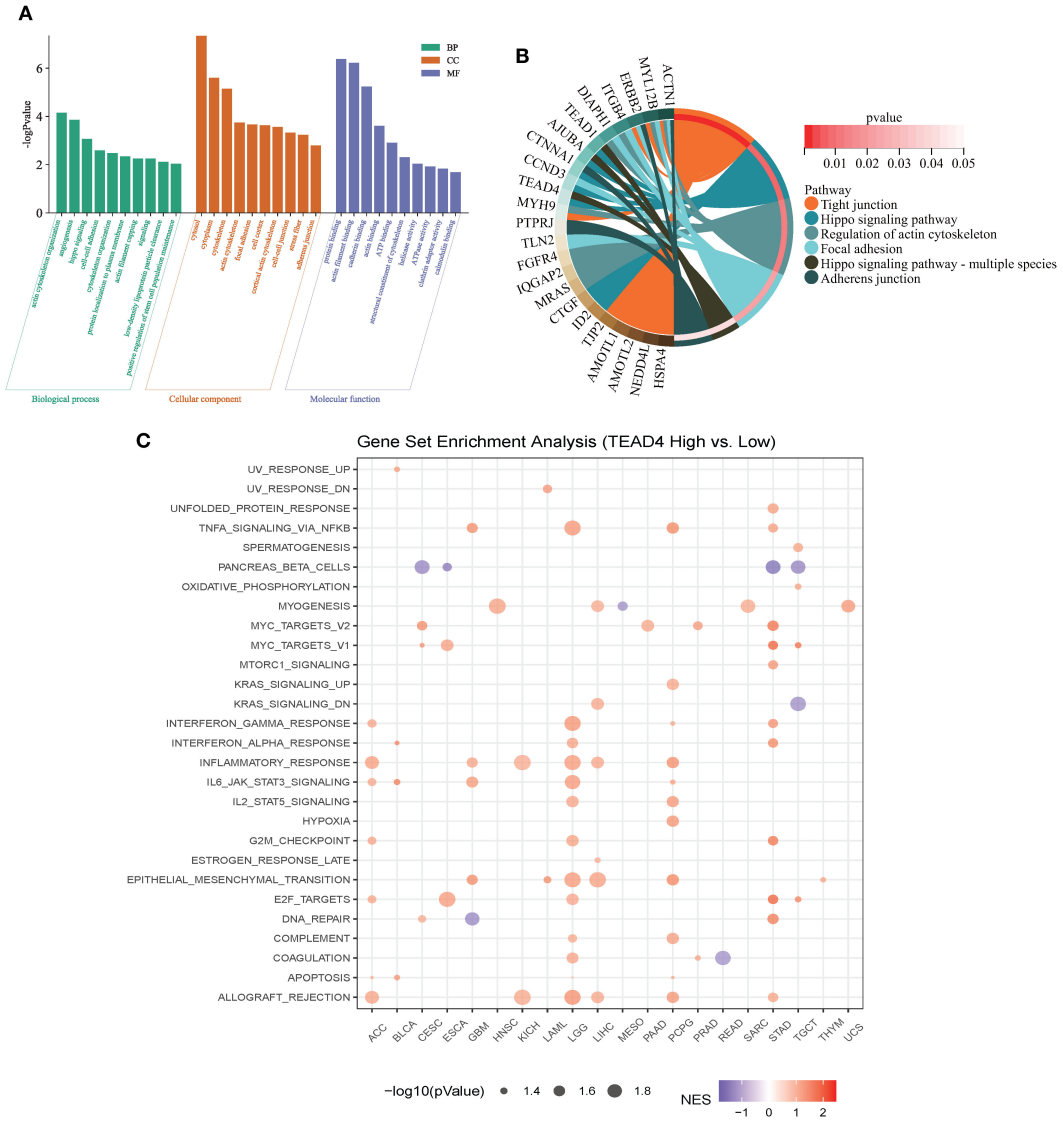
Functional enrichment analysis of TEAD4. **(A)** GO annotation analysis of TEAD4 target genes identified from ENCODE, ChEA3, and hTFtarget databases, with box plots displaying the top 10 biological pathways ranked by enriched gene count. **(B)** Chord diagram visualizing KEGG pathway analysis results of TEAD4 target genes. **(C)** GSEA results demonstrating associations between TEAD4 and HALLMARK pathways across various cancers (*P* < 0.05).

The functional enrichment results provided comprehensive insights into the biological roles of TEAD4. GO annotation revealed that TEAD4 target genes were significantly enriched in biological processes such as transcriptional regulation, angiogenesis, cytoskeletal organization, and cell adhesion. In terms of cellular components, these genes were predominantly localized in the cytoplasm, nucleus, and cytoskeleton. Regarding molecular functions, they were primarily involved in protein synthesis-related activities (P < 0.05) (**Figure 4A**).

KEGG pathway analysis further highlighted significant enrichment in pathways related to tight junctions, the Hippo signaling pathway, and actin cytoskeleton regulation (P < 0.05) (**Figure 4B**). Additionally, gene set enrichment analysis (GSEA) demonstrated that high TEAD4 expression was strongly associated with hallmark processes such as epithelial–mesenchymal transition (EMT), cell cycle regulation, and immune cell infiltration (P < 0.05) (**Figure 4C**).

Together, these findings underscore the multifaceted role of TEAD4 in regulating cancer-related biological functions, including cell adhesion, migration, proliferation, and immune modulation.

### 3.6 Construction and validation of TEAD4 knockdown and overexpression cell line models

Based on the preceding analyses of TEAD4 differential expression, prognostic significance (Cox regression and Kaplan-Meier survival analyses), and GSEA results, a panel of tumor cell lines representing cancers where TEAD4 expression showed strong correlations with clinical outcomes (BLCA, PAAD, KIRC, and LUAD) was selected for subsequent *in vitro* functional validation. It is noteworthy that our prior research had already demonstrated that TEAD4 promotes tumor progression by enhancing EMT in the 5637 bladder cancer cell line; hence, this cell line was directly employed for specific drug sensitivity investigations in the current study.

**Figure 5:**
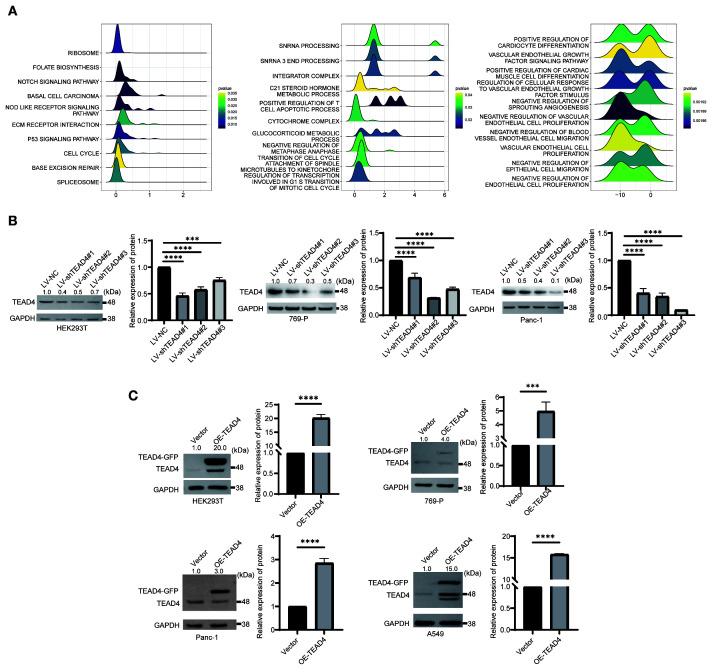
Construction and validation of TEAD4 knockdown and overexpression cell lines. **(A)** GSEA results of TEAD4 in KIRC, LUAD, and PAAD. **(B)** Western Blot detection of TEAD4 knockdown efficiency following lentivirus-mediated TEAD4 knockdown. **(C)** Western Blot analysis of TEAD4 expression levels after TEAD4 overexpression. (***P < 0.001, ****P < 0.0001).

GSEA results in LUAD, KIRC, and PAAD specifically pointed towards TEAD4’s potential regulatory role in cell migration and proliferation-associated pathways (*P* < 0.05) (**Figure 5A**). Given the generally low basal expression of TEAD4 in LUAD tissues, TEAD4 overexpression models were established in the A549 LUAD cell line to investigate its gain-of-function effects. For the Panc-1 (pancreatic cancer) and 769-P (renal cell carcinoma) cell lines, which exhibit moderate endogenous TEAD4 expression, both TEAD4 knockdown and overexpression models were generated to comprehensively evaluate the impact of TEAD4 modulation on tumor cell behavior.

Additionally, to examine how alterations in TEAD4 expression levels affect the regulation of its downstream target genes in a relatively defined system, parallel knockdown and overexpression experiments were conducted in HEK293T cells, which are known for their relatively homogeneous gene expression profiles and ease of transfection. Western blot analysis was performed to confirm the efficiency of TEAD4 protein modulation (both knockdown and overexpression) in all engineered cell lines.

The results successfully demonstrated significant and specific alterations in TEAD4 protein levels as intended, providing a reliable experimental foundation for subsequent functional studies (*P* < 0.05) (**Figures 5B, C**).

### 3.7 TEAD4 promotes cancer cell metastasis *in vitro*

**Figure 6:**
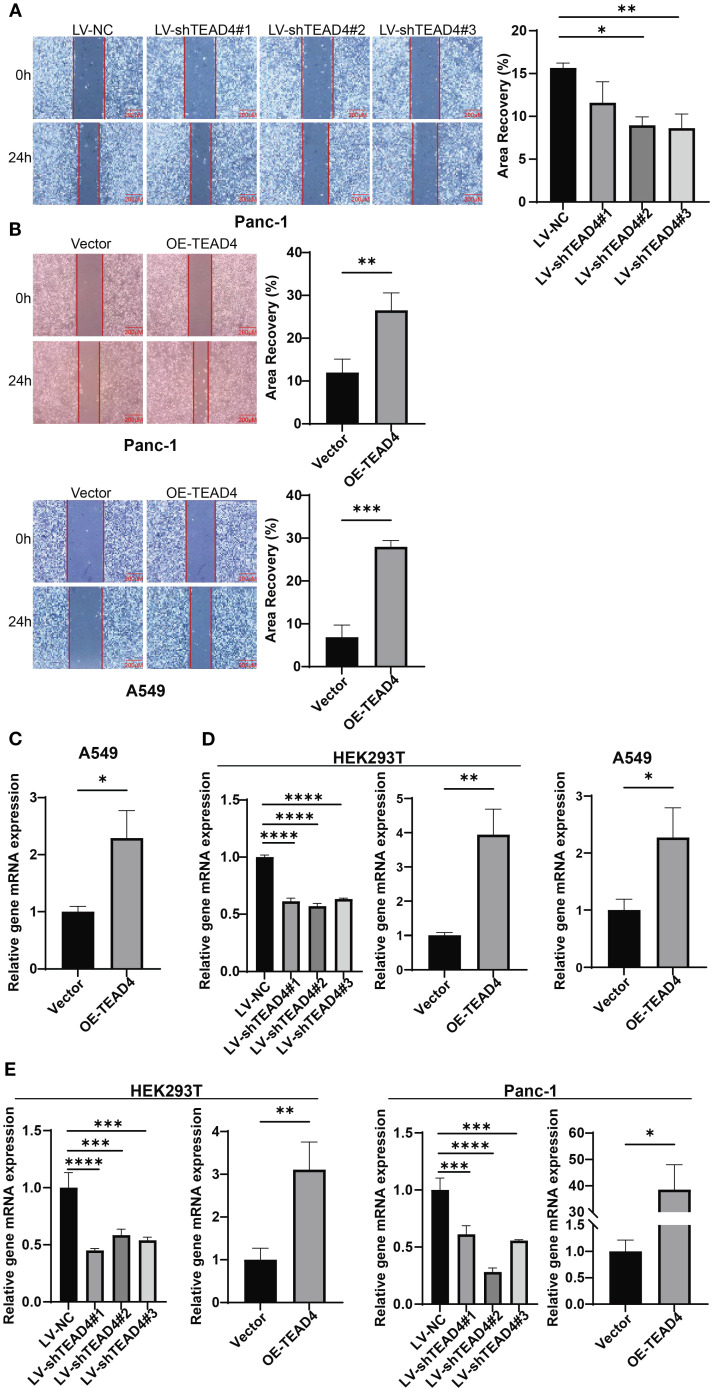
TEAD4 promotes cancer cell metastasis *in vitro*. **(A)** Wound healing assay detection of the effect of TEAD4 knockdown on the migratory ability of Panc-1 cell lines. **(B)** Wound healing assay detection of the effect of TEAD4 overexpression on the migratory ability of Panc-1 and A549 cell lines. **(C)** qRT-PCR analysis of the impact of TEAD4 expression changes on matrix metalloproteinase-9 (MMP-9) gene expression. **(D)** qRT-PCR analysis of the impact of TEAD4 expression changes on Vimentin gene expression. **(E)** qRT-PCR analysis of the impact of TEAD4 expression changes on Snail gene expression. (*P < 0.05, **P < 0.01, ***P < 0.001, ****P < 0.0001).

The wound healing assay was employed to assess the impact of TEAD4 expression on tumor cell migratory capacity. The results demonstrated a consistent positive correlation between TEAD4 expression levels and the migratory potential of cancer cells. Specifically, TEAD4 overexpression significantly enhanced the migration ability of both Panc-1 and A549 cells compared to their respective control cells. Conversely, TEAD4 knockdown markedly inhibited the migration of Panc-1 cells (*P* < 0.05 for all comparisons) (**Figures 6A, B**).

To explore the molecular mechanisms underlying these migratory phenotypes, qRT-PCR analysis of key genes associated with cell migration and EMT was performed. In Panc-1 cells, TEAD4 overexpression led to a significant upregulation of *VIM* (Vimentin) and *SNAI1* (Snail) mRNA expression. Similarly, in A549 cells, TEAD4 overexpression promoted the expression of *SNAI1* and *MMP9* (Matrix Metallopeptidase 9). This regulatory pattern was further validated in HEK293T cells, where TEAD4 overexpression enhanced *VIM* and *SNAI1* expression, while TEAD4 knockdown resulted in their suppression (*P* < 0.05 for all significant changes) (**Figures 6C–E**).

These findings collectively indicate that TEAD4 plays a crucial role in regulating cancer cell migration, at least in part, through the modulation of key migration-related and EMT-associated genes, including *VIM*, *SNAI1*, and *MMP9*.

### 3.8 TEAD4 enhances cancer cell proliferation *in vitro*

**Figure 7:**
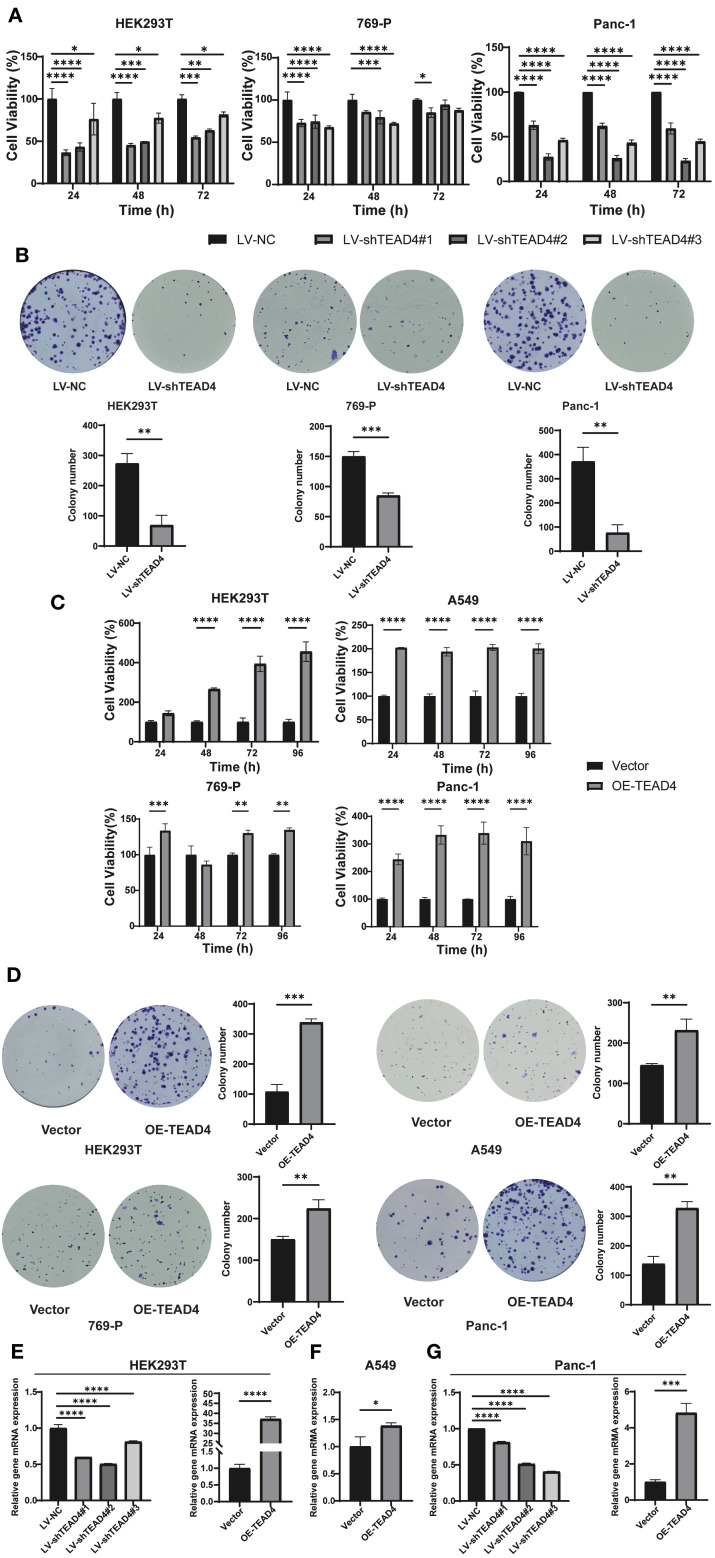
TEAD4 enhances cancer cell proliferation *in vitro.* **(A)** CCK-8 assay assessing the effect of TEAD4 knockdown on the proliferative ability of HEK293T, 769-P, and Panc-1 cell lines. **(B)** Colony formation assessing the effect of TEAD4 knockdown on the proliferation ability of HEK293T, 769-P, and Panc-1 cell lines. **(C)** CCK-8 assay assessing the effect of TEAD4 overexpression on the proliferative ability of HEK293T, A549, 769-P, and Panc-1 cell lines. **(D)** Colony formation assessing the effect of TEAD4 overexpression on the proliferative ability of HEK293T, A549, 769-P, and Panc-1 cell lines. **(E–G)** qRT-PCR analysis of the impact of TEAD4 expression changes on the gene expression of mitotic arrest deficient 2 like 1 (MAD2L1) and RAN binding protein 1 (RANBP1). (*P < 0.05, **P < 0.01, ***P < 0.001, ****P < 0.0001).

The effect of TEAD4 modulation on cancer cell proliferation was evaluated using the CCK-8 assay and colony formation. The results demonstrated that TEAD4 knockdown significantly inhibited the proliferative capacity of HEK293T, Panc-1, and 769-P cells when compared to control cells. Conversely, TEAD4 overexpression significantly promoted cell proliferation in HEK293T, Panc-1, 769-P, and A549 cell lines (*P* < 0.05 for all comparisons) (**Figures 7A–D**).

To investigate the underlying molecular basis for these proliferative effects, qRT-PCR analysis of key cell cycle regulatory genes was performed. The findings revealed that TEAD4 modulates the expression of critical cell cycle regulators (**Figures 7E–G**). Specifically, in both HEK293T and Panc-1 cells, TEAD4 alteration (knockdown or overexpression) significantly affected the expression of *MAD2L1* (Mitotic Arrest Deficient 2 Like 1) (*P* < 0.05) (**Figures 7E, G**). MAD2L1 is a crucial mitotic checkpoint protein that plays a vital role in maintaining chromosomal stability and promoting cancer cell proliferation. Furthermore, TEAD4 overexpression led to a significant upregulation of *RANBP1* (RAN Binding Protein 1) expression in A549 cells (*P* < 0.05) (**Figure 7F**). RANBP1, a nuclear transport factor, is known to facilitate nucleocytoplasmic trafficking and cell cycle progression, thereby driving proliferation.

These results collectively demonstrate TEAD4’s ability to regulate cancer cell proliferation, likely through the modulation of key cell cycle-related genes such as *MAD2L1* and *RANBP1*.

### 3.9 TEAD4 promotes stemness-associated traits in cancer cell

**Figure 8:**
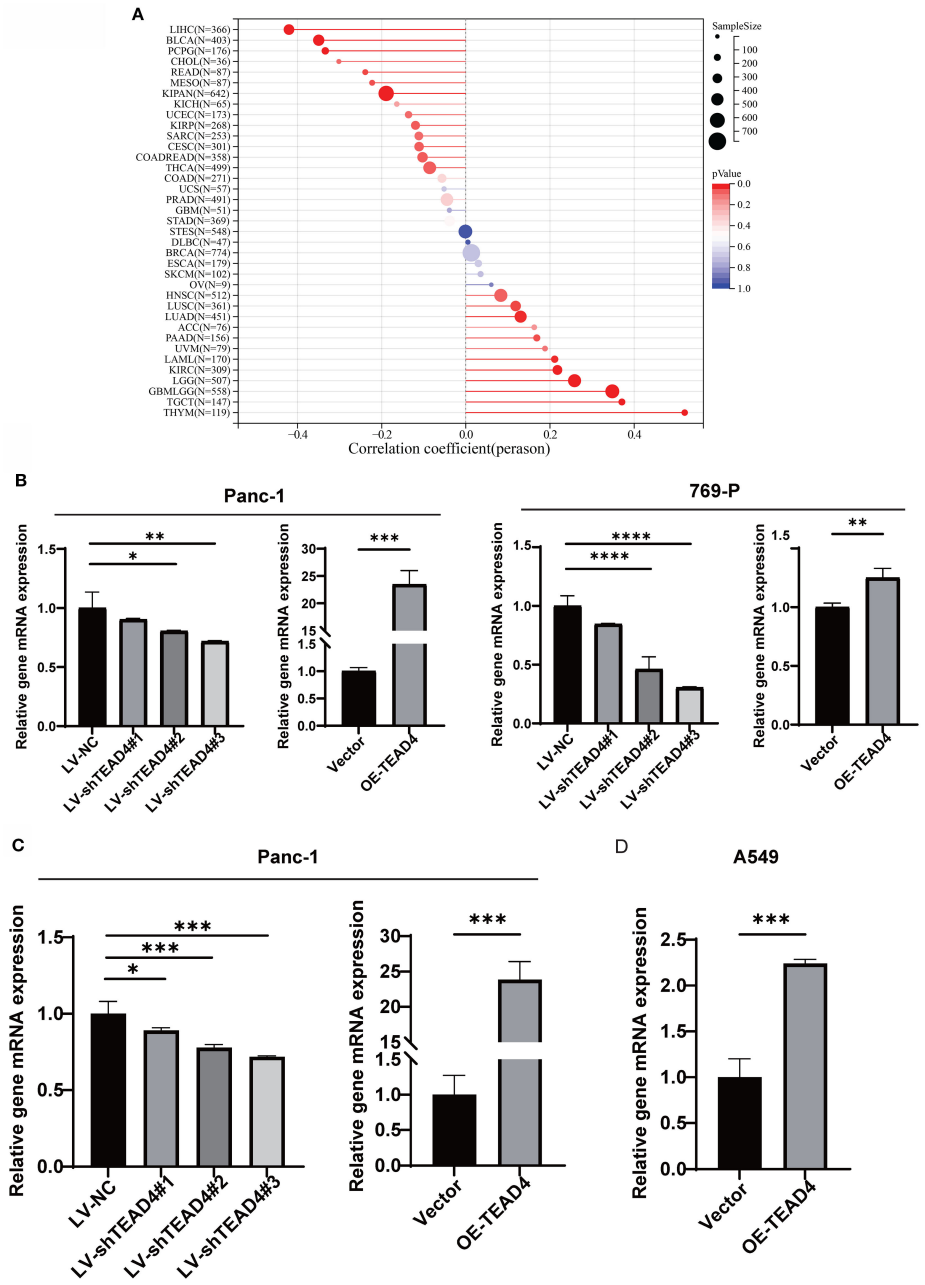
TEAD4 promotes stemness-associated traits in cancer cell. **(A)** Lollipop plot demonstrates the correlation between TEAD4 expression and stemness scores across various tumor tissues. **(B–D)** qRT-PCR analysis evaluating the effects of TEAD4 expression modulation on stemness markers Oct-4, Slug, and Nanog. (*P < 0.05, **P < 0.01, ***P < 0.001, ****P < 0.0001).

The correlation between TEAD4 expression levels and established cancer stemness scores across multiple cancer cell types was first investigated using publicly available data. This analysis revealed a significant positive correlation, suggesting that higher TEAD4 expression is associated with enhanced stem-like properties in cancer cells (**Figure 8A**).

To experimentally validate these correlative findings, the mRNA expression levels of well-recognized stemness markers, including *POU5F1* (Oct-4), *SNAI2* (Slug), and *NANOG*, were quantified in Panc-1, 769-P, and A549 cells following TEAD4 modulation. The qRT-PCR results consistently revealed that TEAD4 expression positively correlated with the expression levels of these stemness markers. Specifically, TEAD4 overexpression upregulated, while knockdown (where applicable) downregulated, the expression of these markers (*P* < 0.05 for significant changes) (**Figures 8B–D**).

These experimental data are in strong agreement with the initial correlation analysis (**Figure 8A**), reinforcing the role of TEAD4 in promoting cancer stemness.

### 3.10 TEAD4 influences genomic instability and epigenetic modifications

To further elucidate TEAD4’s multifaceted role in tumor biology, its potential effects on genomic characteristics, focusing on both genomic stability and epigenetic regulation, were investigated.

**Figure 9:**
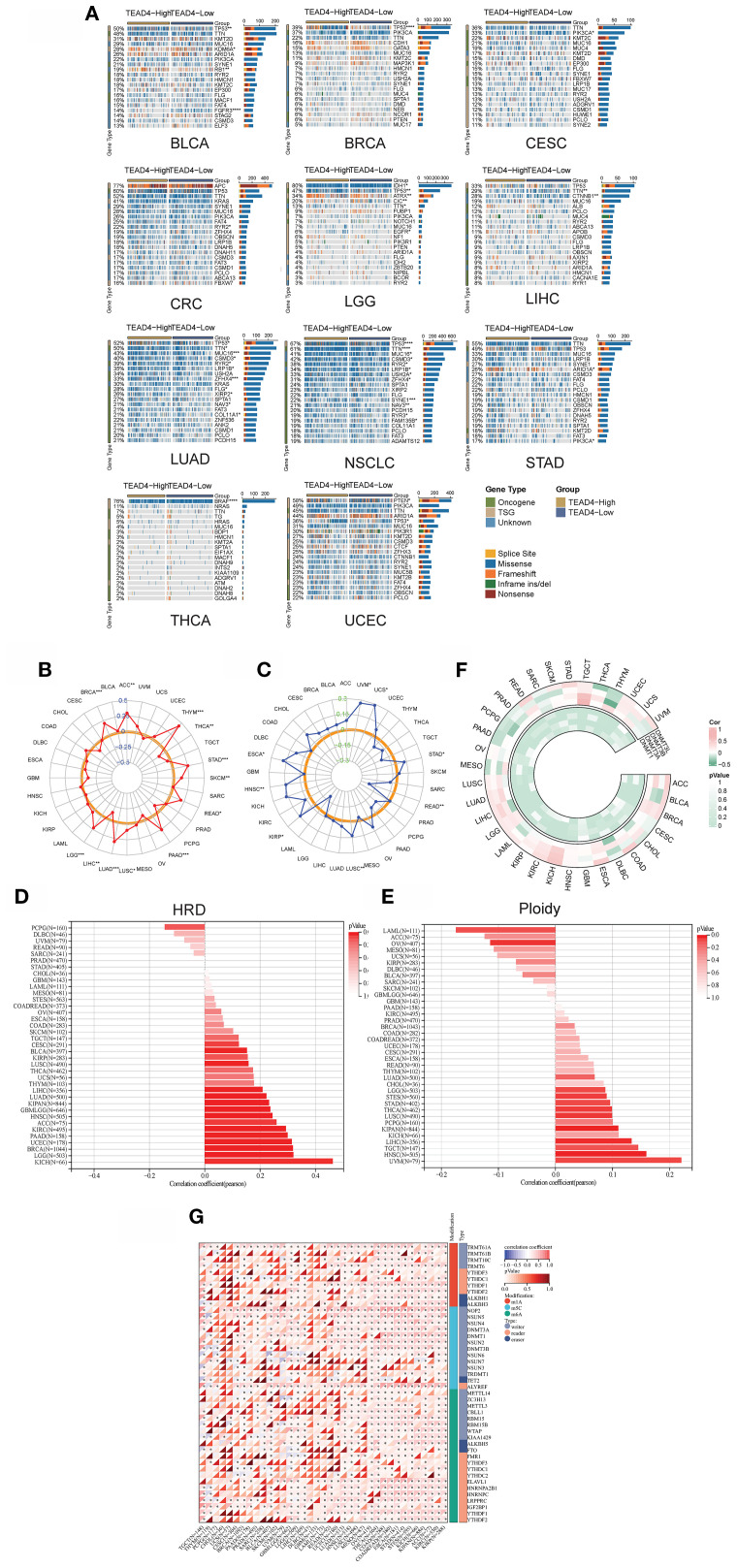
TEAD4 influences genomic instability and epigenetic modifications. **(A)** Analysis of differences in driver gene mutation frequencies between TEAD4 high- and low-expression groups using the CAPIMO database. **(B)** Correlation analysis between TEAD4 expression levels and tumor mutation burden (TMB). **(C)** Correlation analysis between TEAD4 expression levels and microsatellite instability **(D)** Correlation analysis between TEAD4 expression levels and homologous recombination deficiency (HRD). **(E)** Correlation analysis between TEAD4 expression levels and ploidy. **(F)** Expression correlation analysis between TEAD4 and DNA methyltransferase family genes (DNMT1, DNMT3A, DNMT3B, DNMT3L). **(G)** Expression correlation analysis between TEAD4 and RNA modification-related genes (m1A-, m5C-, and m6A-associated genes). (*P < 0.05, **P < 0.01, ***P < 0.001, ****P < 0.0001).

Analysis of the CAMOIP database revealed significant differences in the frequencies of driver gene mutations between TEAD4-high and TEAD4-low expression groups across 11 distinct cancer types (*P* < 0.05) (**Figure 9A**), suggesting a link between TEAD4 expression and the mutational landscape of tumors.

Further correlational analyses demonstrated significant associations between TEAD4 expression levels and multiple established markers of genomic instability. Specifically, TEAD4 expression showed positive correlations with tumor mutational burden (TMB) in 11 cancer types (**Figure 9B**), with microsatellite instability (MSI) status in 6 cancer types (**Figure 9C**), and with homologous recombination deficiency (HRD) scores in 11 malignancies (**Figure 9D**). Moreover, TEAD4 expression was positively associated with gene expression signatures related to ploidy in 6 tumor types (**Figure 9E**). These comprehensive findings suggest that TEAD4 may play extensive regulatory roles in processes related to both the maintenance of genomic stability and the promotion of mutational events across various cancer types.

In terms of epigenetic regulation, co-expression patterns between TEAD4 and key genes encoding DNA methyltransferases (*DNMT1*, *DNMT3A*, *DNMT3B*, *DNMT3L*) were analyzed. The results demonstrated a predominant positive correlation between TEAD4 and *DNMT1* expression in most tumor types. Conversely, negative correlations were generally observed between TEAD4 and *DNMT3A*, *DNMT3B*, and *DNMT3L* expression (*P* < 0.05 for significant correlations) (**Figure 9F**). Notably, high TEAD4 expression also positively correlated with the expression levels of a majority of genes involved in RNA modifications (including those related to m1A, m5C, and m6A modifications) across multiple tumor types (*P* < 0.05) (**Figure 9G**).

These findings suggest that TEAD4 may participate in a complex epigenetic regulatory network that influences both DNA methylation and RNA modification landscapes in cancer.

### 3.11 Potential role of TEAD4 in modulating the tumor immune microenvironment

The broader immune context associated with TEAD4 expression within the tumor mircroenviroment (TME) was first investigated. Utilizing the six established solid tumor immune subtypes defined by Thorsson et al., which carry known therapeutic and prognostic significance, TEAD4’s differential expression across these subtypes was analyzed using the TISIDB database. The results revealed that TEAD4 expression was significantly associated with specific immune subtypes in 16 different tumor types.

**Figure 10:**
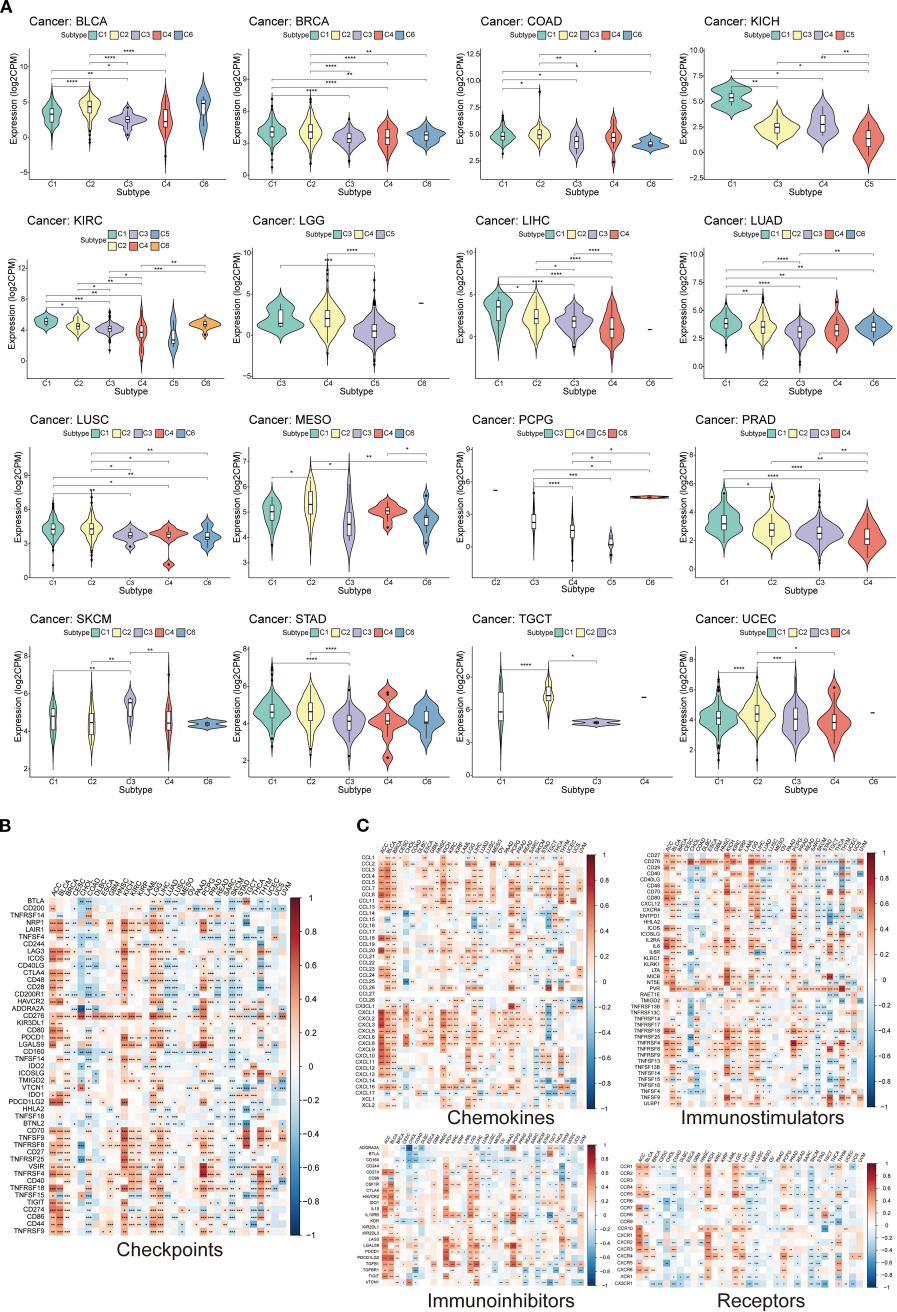
Potential role of TEAD4 in modulating the tumor immune microenvironment. **(A)** Violin plot presenting differential expression analysis of TEAD4 across six immune subtypes (C1-C6: C1, wound healing; C2, IFN-γ dominant; C3, inflammatory; C4, lymphocyte-depleted; C5, immunologically quiet; C6, TGF-β dominant). **(B)** Heatmap illustrating expression correlation analysis between TEAD4 and immune checkpoint genes. **(C)** Heatmap displaying expression correlation analysis between TEAD4 and immune-related genes (chemokines, chemokine receptors, immunostimulators, and immunosuppressors). (*P < 0.05, **P < 0.01, ***P < 0.001, ****P < 0.0001).

Among these associations, TEAD4 exhibited its highest expression levels in the C1 (wound healing) immune subtype across nine cancer types (BLCA, BRCA, KICH, COAD, KIRC, LIHC, LUAD, LUSC, and PRAD). Conversely, in five other cancer types – Pheochromocytoma and Paraganglioma (PCPG), Mesothelioma (MESO), STAD, TGCT, and UCEC – TEAD4 expression peaked in the C2 (IFN-gamma dominant) immune subtype (*P* < 0.05 for significant associations) (**Figure 10A**).

Co-expression relationships between TEAD4 and a panel of critical immune checkpoint genes were further explored. This analysis demonstrated significant positive correlations between TEAD4 expression and the expression of multiple immune checkpoint genes in seven cancer types: ACC, BLCA, KICH, KIRC, LGG, LIHC, and PCPG (*P* < 0.05) (**Figure 10B**).

Recognizing that immune cells often facilitate tumor development and immune evasion through the secretion of cytokines and chemokines, correlations between TEAD4 expression levels and a broad array of immune-related genes, including chemokines, chemokine receptors, immunostimulators, and immunosuppressors, were analyzed. Strong and significant correlations were observed in multiple instances across various cancer types (*P* < 0.05) (**Figure 10C**).

These findings collectively suggest that TEAD4 may play a significant role in regulating the immune status of the TME through diverse molecular mechanisms, potentially participating in immunosuppressive processes and thereby influencing tumor progression and response to immunotherapy across different cancer types.

### 3.12 TEAD4 modulates immune cell composition and activity in TME

**Figure 11:**
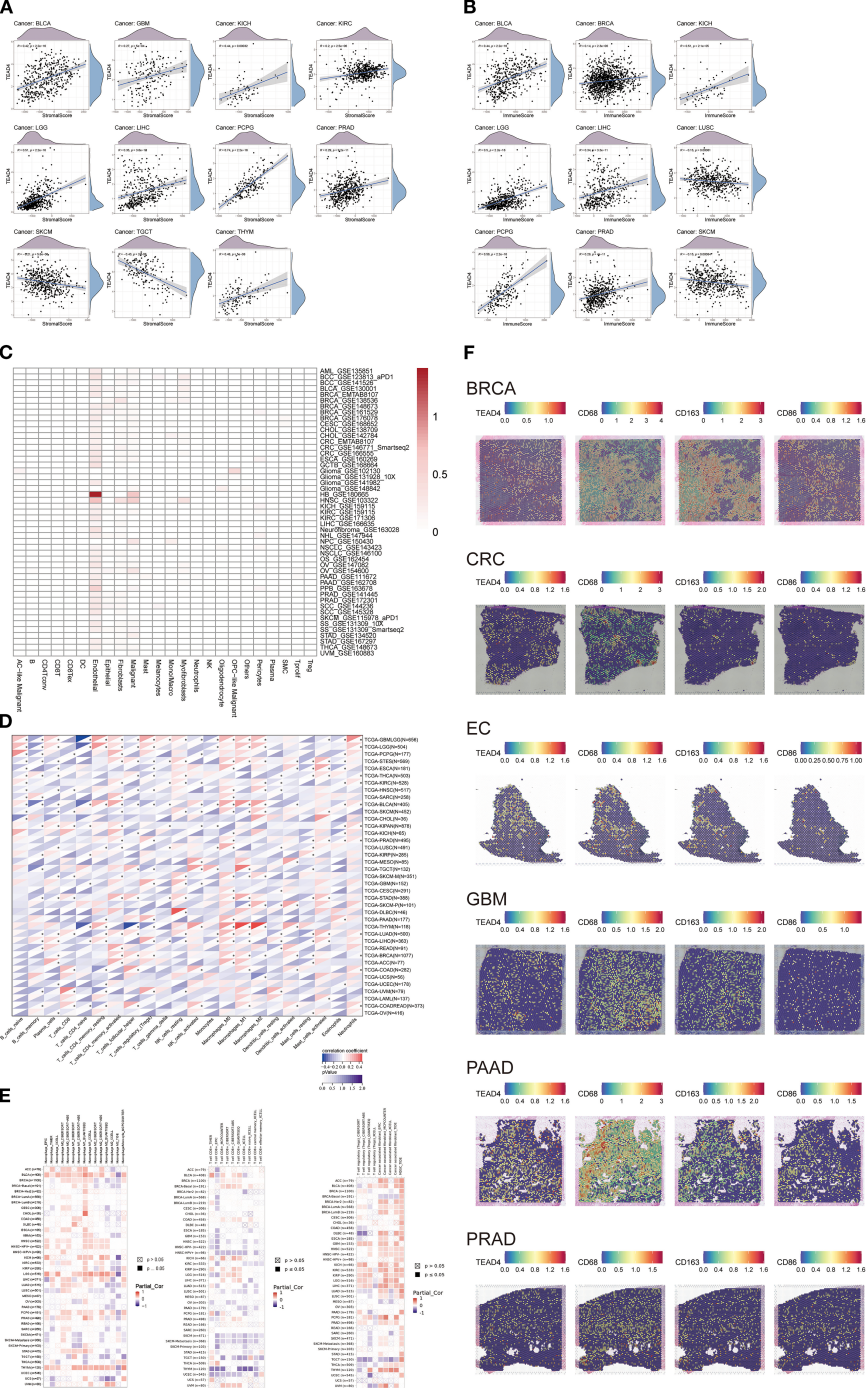
TEAD4 modulates immune cell composition and activity in the TME. **(A, B)** Correlation analyses between TEAD4 mRNA expression and stromal/immune scores in the tumor microenvironment. **(C)** Single-cell RNA sequencing (scRNA-seq) reveals TEAD4 expression patterns across distinct cellular subpopulations. **(D)** CIBERSORT algorithm analysis demonstrates associations between TEAD4 mRNA levels and infiltration abundances of 22 immune cell types. **(E)** Multi-algorithm integration illustrates relationships between TEAD4 expression and specific immune cell subset infiltration (*P < 0.05). **(F)** Spatial transcriptomics visualizes TEAD4-macrophage co-localization patterns within tumor tissues. Each dot represents a specific spatial location on the tissue section. The color gradient from purple to red indicates the level of gene expression: purple denotes the absence of gene expression, while a transition toward red reflects a gradual increase in expression, with red representing the highest level of gene enrichment.

To comprehensively assess the immunomodulatory role of TEAD4 within TME, we conducted integrative analyses to evaluate its influence on immune cell composition and functional dynamics. Using the ESTIMATE algorithm, we identified significant correlations between TEAD4 expression and various TME-related characteristics: stromal scores were positively correlated with TEAD4 expression in nine cancer types—BLCA, GBM, KICH, KIRC, LGG, LIHC, PCPG, PRAD, and THYM—but were negatively correlated in SKCM and TGCT (P < 0.05) (**Figure 11A**).

Immune scores showed positive associations in seven cancers—BLCA, BRCA, KICH, LGG, LIHC, PCPG, and PRAD—and negative associations in LUSC and SKCM (P < 0.05) (**Figure 11B**). These contrasting correlation patterns suggest that TEAD4 may differentially regulate stromal and immune components across tumor types.

To further delineate TEAD4 expression at the single-cell level, we analyzed single-cell RNA sequencing (scRNA-seq) data from the GEO database. The analysis revealed that TEAD4 expression was highest in endothelial cells, indicating a cell-type-specific expression pattern (**Figure 11C**). We next applied the CIBERSORT algorithm to systematically evaluate the correlation between TEAD4 mRNA expression and the infiltration levels of 22 immune cell subsets (**Figure 11D**). In parallel, TIMER2.0 multi-algorithm analysis identified significant associations between TEAD4 expression and specific immune cell populations (**Figure 11E**). Notably, TEAD4 expression was negatively correlated with CD8^+^ T cell infiltration in the majority of cancer types, while showing positive correlations with regulatory T cells (Tregs). Additionally, TEAD4 expression was positively associated with infiltration of macrophage subsets, including M0, M1, and M2 macrophages.

To validate these associations, spatial transcriptomics analysis was performed, which visually confirmed the co-localization of TEAD4 with macrophage markers - CD68, CD163, and CD86 - in tumor tissue specimens (**Figure 11F**). The spatial transcriptomic map reveals that each dot corresponds to a specific spatial location within the tissue section. Purple dots indicate the absence of gene expression at a given location, while other colors represent varying levels of gene expression. Notably, regions with high TEAD4 expression show significant spatial overlap with the distribution of macrophage markers. This finding visually confirms the close interaction between TEAD4 and tumor-associated macrophages within the tumor microenvironment at a spatial resolution, providing important morphological evidence for further investigation into the underlying molecular mechanisms.

Collectively, these multi-dimensional analyses demonstrate that TEAD4 plays a complex and context-dependent role in modulating immune cell infiltration, phenotype, and spatial architecture within the TME, thereby potentially influencing tumor immunity and progression.

### 3.13 Association of TEAD4 expression with clinical treatment efficacy

**Figure 12:**
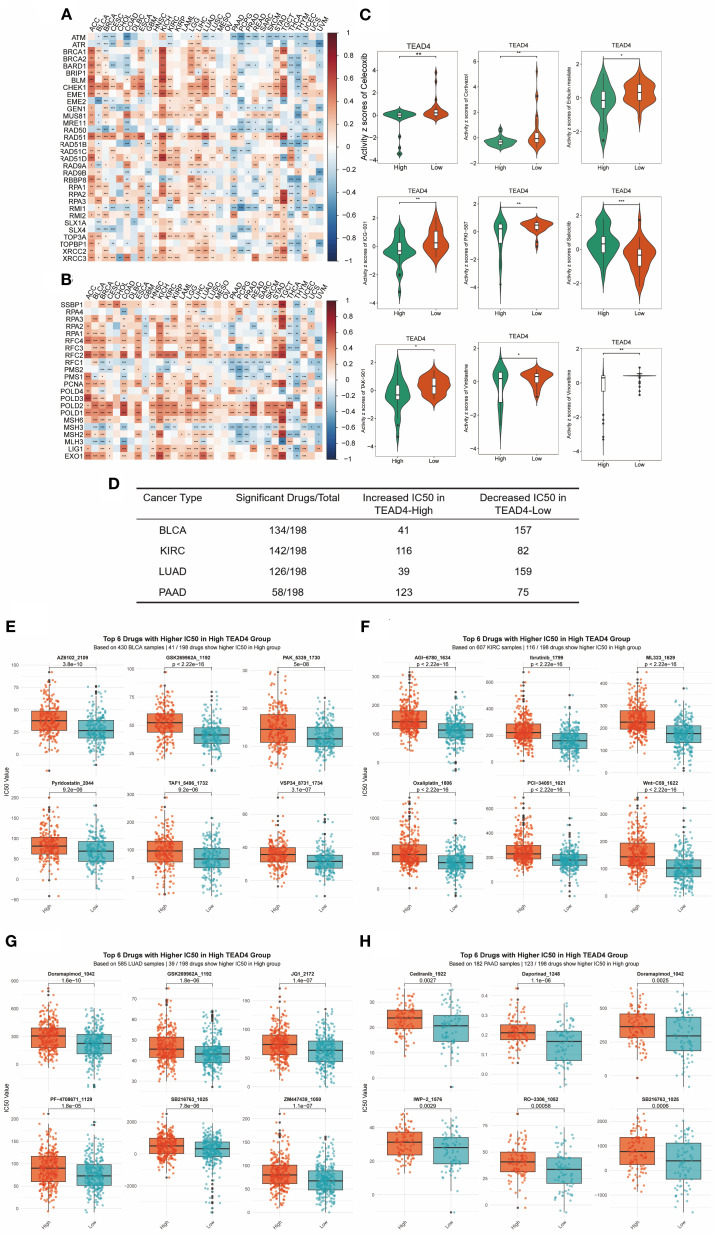
Association of TEAD4 expression with clinical treatment efficacy. **(A)** Heatmap displaying co-expression patterns between TEAD4 and homologous recombination repair (HRR)-related genes across tumors. **(B)** Heatmap illustrating co-expression relationships of TEAD4 with mismatch repair (MMR)-associated genes in malignancies. **(C)** CellMiner database analysis demonstrating differential drug sensitivity between TEAD4-high and TEAD4-low expression groups. **(D)** Summary table showing the number of chemotherapeutic agents (out of 198 drugs in the GDSC database) whose IC_50_ values were significantly affected by TEAD4 expression across four cancer types (BLCA, LUAD, KIRC, PAAD). For each cancer type, the table indicates both the total number of drugs influenced and their distribution into two categories: drugs with lower IC_50_ in the TEAD4-high group (increased sensitivity) and drugs with higher IC_50_ in the TEAD4-high group (reduced sensitivity). **(E–H)** Boxplots showing the top six drugs (ranked by the smallest P-values) with significant IC_50_ differences between TEAD4-high and TEAD4-low groups in BLCA, KIRC, LUAD, and PAAD, respectively. Higher IC_50_ values in the TEAD4-high group indicate reduced sensitivity. Statistical significance is denoted as *P < 0.05, **P < 0.01, ***P < 0.001, **P < 0.0001.

We investigated the association between TEAD4 expression and key DNA repair mechanisms to explore its potential role in therapeutic resistance. Our analysis revealed consistent positive correlations between TEAD4 and multiple DNA repair pathways. Specifically, TEAD4 expression exhibited significant co-expression with 34 HRR - related genes across various tumor types, with particularly strong associations observed in ACC, BLCA, COAD, KICH, LUAD, READ, and STAD (P < 0.05) (**Figure 12A**).

Similarly, TEAD4 showed positive correlations with mismatch repair (MMR) genes in a broad range of cancers, including BLCA, BRCA, ESCA, HNSC, KICH, LAML, LGG, LIHC, LUAD, LUSC, STAD, TGCT, THYM, and UCEC (P < 0.05) (**Figure 12B**). These findings suggest that TEAD4 may contribute to the maintenance of cancer cell stemness and resistance to radiotherapy, potentially through the enhancement of DNA repair capacity.

To further explore TEAD4’s clinical relevance, we assessed its relationship with chemotherapeutic drug sensitivity using data from the CellMiner database. Among 33 FDA-approved anticancer drugs analyzed, nine compounds demonstrated significantly different responses between TEAD4-high and TEAD4-low expression groups (P < 0.05) (**Figure 12C**). Notably, eight of these drugs exhibited reduced sensitivity in TEAD4-high tumors, indicating a potential role for TEAD4 in mediating chemoresistance to multiple agents.

Based on the GDSC database, we analyzed four cancer types (BLCA, KIRC, LUAD, and PAAD) in which TEAD4 was experimentally validated to promote oncogenesis *in vitro*, and compared the IC50 values of 198 chemotherapeutic drugs between TEAD4-high and TEAD4-low groups (**Figure 12D**). In BLCA, 134 drugs showed differential IC50 values: 41 drugs exhibited higher IC50 in the TEAD4-high group, while 93 drugs showed higher IC50 in the TEAD4-low group, indicating mixed effects on drug sensitivity. A similar distribution was observed in LUAD. In contrast, in KIRC and PAAD, the majority of drugs displayed increased IC50 in the TEAD4-high group, suggesting a stronger association with chemotherapy resistance (**Figures 12E–H**).

Together with CellMiner results, which showed a general association of TEAD4-high tumors with reduced drug sensitivity across multiple FDA-approved agents, these findings suggest that TEAD4 influences drug response in a cancer type– and drug-specific manner.

Collectively, these results identify TEAD4 as a dual regulator of therapeutic resistance in cancer. Mechanistically, TEAD4 appears to promote radioresistance by activating DNA repair pathways, while concurrently driving chemoresistance by modulating cellular drug responsiveness. These insights not only enhance our understanding of TEAD4’s role in treatment resistance but also provide a compelling rationale for the development of TEAD4-targeted combination therapies to overcome resistance to both radiation and chemotherapeutic interventions.

### 3.14 Construction of a predictive modeling based on TEAD4 target genes

In our preliminary analysis, we identified six tumor types that exhibited significant TEAD4-associated survival differences based on Kaplan–Meier survival curves, which were selected for further investigation. We subsequently integrated 209 potential TEAD4 target genes derived from three transcription factor databases: ENCODE, ChEA3, and hTFtarget. To enhance model accuracy, we retained only those target genes that showed an absolute correlation coefficient > 0.1 with TEAD4 expression. A cross-tumor comparative analysis narrowed this list down to 37 key target genes, which formed the basis of our prediction model.

**Figure 13:**
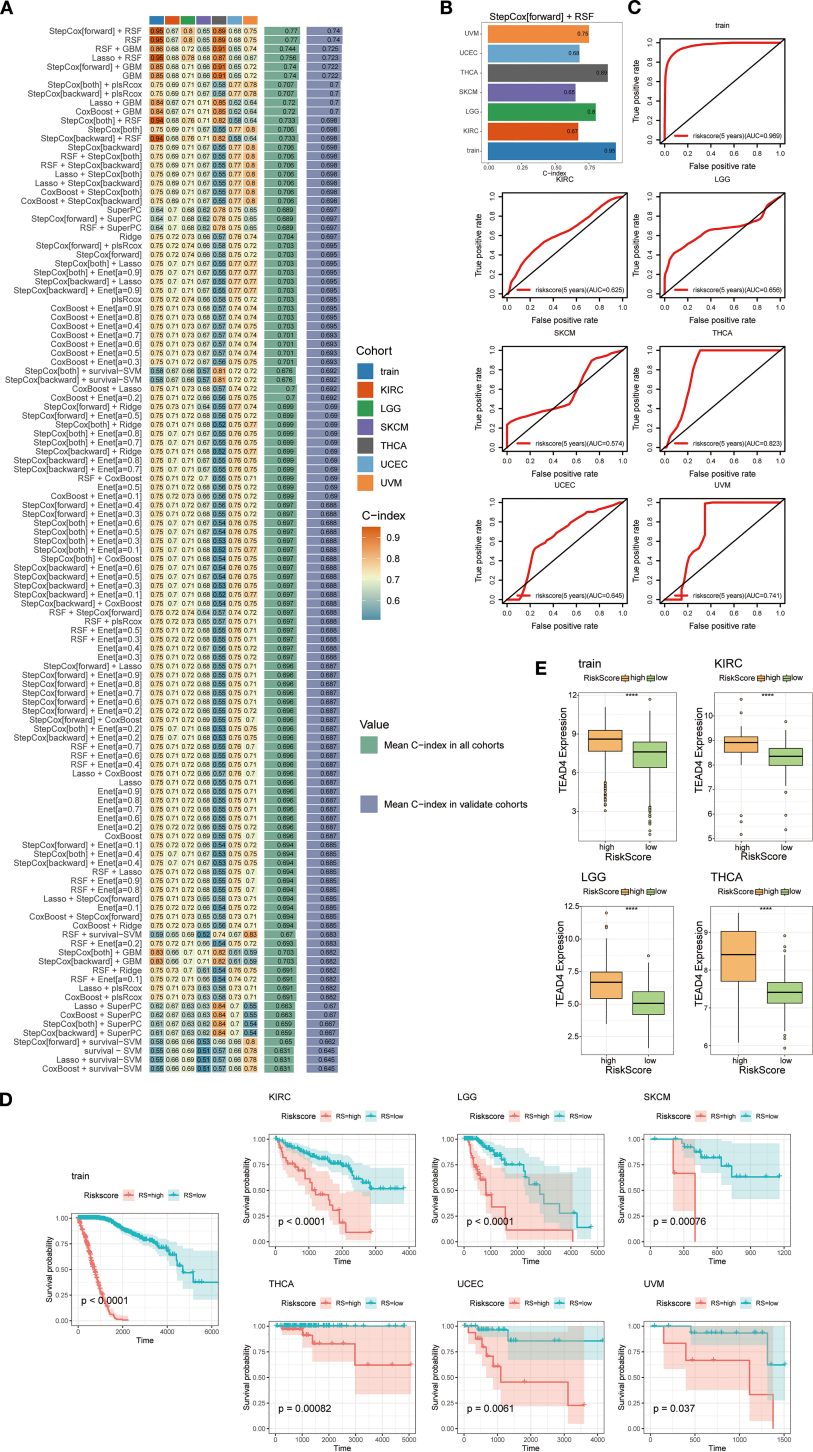
Construction of a predictive modeling based on TEAD4 target genes. **(A)** Performance evaluation of 101 prediction models constructed using a 10-fold cross-validation framework, with C-indices calculated for each model across all validation datasets. **(B)** Bar plot demonstrating the C-index of the optimal prediction model. **(C)** ROC curves evaluating survival prediction accuracy based on risk score stratification. **(D)** Kaplan-Meier survival curves revealing the association between high/low risk scores and overall survival (OS) in cancer patients. **(E)** Box plots showing differential TEAD4 expression between high- and low-risk score groups. (****P < 0.0001).

During the model construction phase, we evaluated the performance of 101 machine learning algorithms, ultimately identifying the combined Stepwise Cox regression (StepCox[forward]) and Random Survival Forest (RSF) approach as the most effective (**Figures 13A, B**). This integrative model utilized the 37 TEAD4-associated target genes as input variables to generate individualized patient risk scores.

Model performance was assessed using receiver operating characteristic (ROC) curve analysis, and the results demonstrated high predictive accuracy. The concordance index (c-index) for most tumor types exceeded 0.6, with several models achieving values above 0.8, underscoring the robustness of the predictive system (**Figure 13C**).

Further validation via Kaplan–Meier survival analysis confirmed the clinical relevance of the model. Stratification of patients into high-risk and low-risk groups based on calculated risk scores revealed significant differences in survival outcomes between the groups (**Figure 13D**). Notably, TEAD4 expression levels were significantly higher in the high-risk group compared to the low-risk group, consistent with our hypothesis and reinforcing the biological plausibility and clinical utility of the model (**Figure 13E**).

This predictive framework not only serves as a survival prediction tool rooted in the TEAD4 regulatory network, but also offers novel insights into the functional mechanisms of TEAD4 across diverse cancer types. Moreover, it lays a theoretical foundation for the development of personalized therapeutic strategies targeting TEAD4 in the context of precision oncology.

## 4 Discussion

Cancer is fundamentally characterized by uncontrolled cellular proliferation and genomic instability, representing a major global health burden with persistent therapeutic challenges (31). A key limitation of conventional anticancer therapies lies in their suboptimal selectivity, which impairs the ability to effectively distinguish malignant cells from their normal counterparts. This non-specificity frequently results in systemic toxicity, compromising patient quality of life and limiting therapeutic efficacy. Therefore, a deeper understanding of the molecular alterations that differentiate cancerous from normal cells is essential for the rational development of targeted therapeutic strategies. Systematic identification of tumor-specific molecular vulnerabilities will facilitate the design of precision therapies with improved efficacy and minimized off-target effects (32).

The advent of molecularly targeted therapies has marked a paradigm shift in oncology. Notable clinical successes include small-molecule tyrosine kinase inhibitors (TKIs) targeting the epidermal growth factor receptor (EGFR) pathway, such as Gefitinib, Erlotinib, and Lapatinib. These agents selectively inhibit aberrant EGFR signaling, a key oncogenic driver in various malignancies. Erlotinib and Lapatinib have been approved for the treatment of advanced non-small cell lung cancer (NSCLC) with sensitizing EGFR mutations, while Lapatinib has also demonstrated efficacy in HER2-positive breast cancers (33). Importantly, these agents highlight a core advantage of targeted therapy: the ability to exploit a single dysregulated molecular axis across multiple tumor types, thereby streamlining drug development and expanding clinical indications.

The Hippo signaling pathway, evolutionarily conserved across species, is a central regulator of organ size, cell proliferation, and tissue homeostasis. In physiological conditions, core Hippo kinases phosphorylate and inactivate the transcriptional co-activators YAP (Yes-associated protein) and TAZ (transcriptional coactivator with PDZ-binding motif), resulting in their cytoplasmic sequestration and/or degradation. This inactivation prevents the transcription of genes involved in proliferation, apoptosis, migration, and differentiation (34). In contrast, dysregulation of the Hippo pathway - commonly observed in cancer - leads to nuclear accumulation of YAP/TAZ, which, through interaction with TEAD family transcription factors (TEAD1 - 4), activates oncogenic transcriptional programs that drive tumor initiation and progression (34).

Our pan-cancer analysis identifies TEAD4 as a central effector of Hippo pathway dysregulation, mediating its oncogenic transcriptional outputs. Recognizing the lack of a comprehensive, systematic understanding of TEAD4’s roles across diverse cancer types, we employed an integrative bioinformatics approach to delineate its molecular landscape, regulatory networks, and clinical relevance. By integrating multi-omics data - including genomic, transcriptomic, and proteomic profiles with clinical outcome information, we constructed a comprehensive framework for TEAD4 regulation across human malignancies (9, 15, 35).

TEAD4 was found to be significantly upregulated in the majority of tumor types compared to normal tissues. Notably, its expression increased with advancing tumor stages, suggesting a progressive role in malignant transformation and aggressiveness. Further, elevated TEAD4 levels were consistently associated with poorer clinical outcomes across multiple survival metrics, including OS, DSS, and PFI, underscoring its potential as a broadly applicable prognostic biomarker.

To elucidate the molecular underpinnings of TEAD4’s oncogenic roles, we performed functional enrichment analyses, including GO, KEGG pathway analysis, and GSEA. To validate these bioinformatic insights, we selected four representative cell lines for experimental investigation: 769-P and Panc-1 cells were chosen due to the paucity of existing TEAD4 studies in these models; A549 was included based on our observation that, despite low baseline TEAD4 levels in LUAD, its overexpression was strongly associated with poor prognosis; and HEK293T served as a tool cell line for mechanistic investigations involving gene overexpression and knockdown.

Functional assays demonstrated that TEAD4 promotes malignant phenotypes, including increased cellular migration, invasion, and proliferation - hallmarks of cancer progression. Additionally, TEAD4 was identified as a key regulator of cancer stemness, a property critical for tumorigenesis, therapeutic resistance, and metastatic recurrence (8, 15, 36). Although assays such as wound healing, CCK-8 and colony formation have been previously employed to assess TEAD4 function, our experiments add novelty by validating TEAD4’s roles in less-explored tumor types (LUAD, PAAD, KIRC) and directly linking phenotypic changes to transcriptomic targets highlighted in our pan-cancer analyses. These results provide integrative validation that bridges large-scale computational predictions with targeted *in vitro* evidence, offering a complementary perspective to prior TEAD4 studies.

Interestingly, although bulk TCGA data showed reduced TEAD4 mRNA in LUAD, our functional assays revealed clear oncogenic effects upon TEAD4 overexpression in LUAD cells. This discrepancy likely reflects tumor heterogeneity, subtype-specific expression, and post-transcriptional regulation, emphasizing the context-dependent role of TEAD4 in LUAD biology.

To further investigate TEAD4’s role in genomic instability and epigenetic regulation, we analyzed cancer driver gene mutation frequencies in TEAD4-high vs. TEAD4-low expression groups. The TEAD4-high cohort exhibited significantly elevated frequencies of driver gene mutations (P < 0.05), suggesting a possible link between TEAD4 expression and genomic instability. Furthermore, correlation analyses revealed significant associations between TEAD4 expression and epigenetic regulators, indicating its involvement in modulating chromatin states and transcriptional programs.

TEAD4 acts as a central transcriptional hub integrating multiple oncogenic circuits. Upstream, it cooperates with Hippo pathway effectors YAP/TAZ to activate tumor-promoting transcription. Downstream, TEAD4 regulates EMT and stemness genes (e.g., VIM, SNAI1/2, NANOG) and cell cycle regulators (MAD2L1, RANBP1), thereby enhancing proliferation, plasticity, and genomic stability. TEAD4 also correlates with DNA repair pathways, suggesting a role in therapeutic resistance, and with immune checkpoints and macrophage polarization, implicating it in immune evasion. Collectively, these results indicate that TEAD4 integrates Hippo-YAP/TAZ signaling with transcriptional programs governing EMT, stemness, DNA repair, and immune modulation.

We also explored TEAD4’s influence on the TME. TEAD4 expression was enriched in immune subtypes C1 (wound healing/immunosuppressive) and C2 (IFN-γ-dominant/inflammatory), suggesting subtype-specific immunomodulatory roles. Correlations were identified between TEAD4 and key immune factors, including checkpoint molecules, chemokines, and immunomodulators. Using the ESTIMATE algorithm, TEAD4 was found to positively correlate with stromal scores (P < 0.01) and negatively with immune cell infiltration (P < 0.05), indicating its potential role in immune exclusion.

Simultaneously, it is also important to place TEAD4 in the context of the broader TEAD transcription factor family (TEAD1 - 4). While all TEAD proteins share a conserved TEA DNA-binding domain and cooperate with YAP/TAZ in oncogenic transcription, their roles in tumor immunity appear distinct. TEAD1 and TEAD3 have been implicated in promoting proliferation and survival in several cancers, but direct evidence for immune regulation is limited (37–39). TEAD2 contributes to stemness and has been suggested to indirectly influence immune evasion through YAP/TAZ signaling (40–42). By contrast, TEAD4 shows the most consistent associations with immune checkpoint expression, macrophage polarization, and T cell exclusion across cancers (13, 43). These observations suggest functional redundancy among TEAD family members, yet highlight TEAD4 as a predominant regulator of the tumor immune microenvironment, warranting future comparative studies.

Single-cell transcriptomic analysis revealed high TEAD4 expression in tumor-associated endothelial cells, implicating its involvement in angiogenesis. TEAD4 expression was inversely correlated with CD8^+^ T cell infiltration and positively correlated with regulatory T cells, suggesting a role in establishing an immunosuppressive TME. Its association with M0/M1/M2 macrophage infiltration, supported by spatial transcriptomics, further indicates a role in macrophage polarization.

Additionally, TEAD4 was significantly associated with DDR pathways. TEAD4-high tumors showed enhanced HRR and MMR gene expression, indicating a potential role in radio-resistance. Our analyses further extend the link between TEAD4 and therapy resistance. Drug sensitivity analyses using the CellMiner database demonstrated that high TEAD4 expression correlated with resistance to multiple chemotherapeutic agents, highlighting its role in modulating treatment response and resistance mechanisms. While CellMiner data indicated a general association between high TEAD4 expression and reduced drug sensitivity, GDSC results revealed a more context-dependent pattern. In BLCA and LUAD, TEAD4-high tumors showed increased sensitivity to subsets of agents, whereas in KIRC and PAAD, TEAD4-high expression was more consistently linked to resistance. These divergent trends likely reflect tissue-specific transcriptional programs, co-mutation backgrounds, or distinct drug mechanisms. Taken together, these results suggest that TEAD4 does not act as a uniform resistance marker but modulates chemotherapy response in a cancer type– and drug-specific manner. Future studies using patient-derived models will be essential to clarify the causal role of TEAD4 in therapeutic resistance.

Building on these findings that implicate TEAD4 in DNA repair and therapy resistance, it is important to consider the therapeutic potential of directly targeting TEAD4 and its co-regulatory circuits. Yet, direct inhibition of transcription factors is difficult, and TEAD family redundancy plus homeostatic functions raise further challenges. Current strategies mainly disrupt YAP/TAZ - TEAD4 interactions, with small molecules or peptides targeting the palmitoylation pocket or binding interfaces showing promising preclinical efficacy (44, 45). Nonetheless, issues remain, including compensatory activity from other TEADs, context-dependent effects complicating patient selection, and potential toxicity from long-term inhibition. Future work should emphasize selective inhibitors, predictive biomarkers such as TEAD4 target signatures, and rational combination therapies. Although still early, the expanding pipeline of YAP/TAZ - TEAD inhibitors supports the translational potential of stargeting TEAD4. Thus, while TEAD4 - directed therapies are conceptually promising, careful evaluation of efficacy, safety, and patient selection will be critical before clinical translation.

Overall, while TEAD4-directed therapy is still at a preclinical stage, accumulating mechanistic insights and ongoing drug development provide cautious optimism for eventual clinical translation.

### 4.1 Limitations

Despite these strengths, our study has notable limitations. First, our functional validation was confined to *in vitro* assays, and *in vivo* confirmation in xenograft or genetically engineered mouse models is still required to firmly establish the translational impact of TEAD4 modulation. Second, many of the reported associations, particularly those involving immune subtypes, checkpoint molecules, and genomic instability, were based on correlation analyses of bulk and single-cell datasets. Such correlations are informative but do not prove causation and should be regarded as hypothesis-generating. Future studies employing animal models and perturbation-based approaches will be essential to validate these findings and to delineate the causal mechanisms underlying TEAD4-driven tumor progression and immune regulation.

## 5 Conclusion

In this comprehensive pan-cancer analysis, we demonstrated that TEAD4 is aberrantly expressed across multiple malignancies and consistently associated with poor clinical outcomes. By integrating multi-omics profiling with functional assays, we delineated TEAD4’s multifaceted roles in driving tumor progression, stemness, genomic instability, immune modulation, and therapy resistance. These findings not only broaden the current understanding of TEAD4 biology but also underscore its promise as a prognostic biomarker and potential therapeutic target. Nevertheless, *in vivo* validation and clinical studies will be essential to confirm its translational relevance. Ultimately, such efforts will be critical to bridge molecular insights into the development of safe and effective TEAD4-directed precision therapies.

## Data Availability

The datasets presented in this study can be found in online repositories. The names of the repository/repositories and accession number(s) can be found in the article/**Supplementary Material**.

## Glossary

ACC: Adrenocortical carcinoma
BLCA: Bladder cancer
BRCA: Breast cancer
CESC: Cervical cancer
CHOL: Bile duct cancer
COAD: Colon cancer
CNV: Copy number variation
CRC: Colorectal cancer
DLBC: Large B-cell lymphoma
DMEM: Dulbecco’s modified eagle medium
DSS: Disease free survival
EMT: Epithelial-mesenchymal transition
ESCA: sophageal cancer
ESCC: sophageal squamous cell carcinoma
GBM: Glioblastoma multiforme
GO: Gene ontology
GSEA: Gene set enrichment analysis
HCC: epatocellular carcinoma, HNSC, Head and neck squamous cell carcinoma
HRD: Homologous
KEGG: Kyoto encyclopedia of genes and genomes
KICH: Kidney chromophobe
KIRC: Kidney clear cell carcinoma
KIRP: Kidney papillary cell carcinoma
LAML: Acute myeloid leukemia
LIHC: Liver cancer
LGG: Lower grade glioma
LUAD: Lung adenocarcinoma
LUSC: Lung squamous cell carcinoma
MAD2L1: Mitotic arrest deficient 2 like 1
MMP-2: Matrix metalloproteinase 2
MMP-9: Matrix metalloproteinase 9
MSI: Microsatellite instability
OS: Overall survival
OV: Ovarian cancer
PAAD: Pancreatic cancer
PCNA: Proliferating cell nuclear antigen
PCPG: Pheochromocytoma &
PEI: Polyethyleneimine
PFI: Progression free interval
PRAD: Prostate cancer
PVDF: Polyvinylidene fluoride;
qRT-PCR: Quantitative reverse transcription PCR
RANBP1: RAN binding protein 1
READ: Rectal cancer
RIPA: Radio immunoprecipitation assay buffer
SARC: Sarcoma
SKCM: Skin cutaneous melanoma
STAD: Stomach cancer
TEAD4: TEA domain transcription factor 4
TCGA: The cancer genome atlas
TGCT: Testicular cancer
THCA: Thyroid cancer
THYM: Thymoma
TME: Tumor microenvironment
TMB: Tumor mutation burden
UCEC: Endometrioid cancer
UCS: Uterine carcinosarcoma
UVM: Uveal melanoma
