# Supplementary material for: Integrative analysis identifies TEAD4 as a universal prognostic biomarker in human cancers
Source: Front Immunol. 2025 Sep 30;16:1688563. doi: 10.3389/fimmu.2025.1688563 (PMC12518352; doi:10.3389/fimmu.2025.1688563)
Supplement: Supplementary file 1 [file Table1.docx]

**Table 1, Single-cell transcriptomic sample information**

| **Cohort** | **Data set** | **Sample size** |
| --- | --- | --- |
| LAML | GSE135851 | 8 |
| BCC | GSE123813 | 86 |
|  | GSE141526 | 8 |
| BLCA | GSE130001 | 2 |
| BRCA | EMTAB8107 | 18 |
|  | GSE138536 | 1902 |
|  | GSE148673 | 13 |
|  | GSE161529 | 69 |
|  | GSE176078 | 26 |
| CESC | GSE168652 | 2 |
| CHOL | GSE138709 | 8 |
|  | GSE142784 | 2 |
| CRC | EMTAB8107 | 7 |
|  | GSE146771 | 20 |
|  | GSE166555 | 25 |
| ESCA | GSE160269 | 128 |
| GCTB | GSE168664 | 1 |
| Glioma | GSE102130 | 4058 |
|  | GSE131928 | 2 |
|  | GSE141982 | 4 |
|  | GSE148842 | 43 |
| HB | GSE180665 | 7 |
| HNSC | GSE103322 | 5901 |
| KICH/KIRC | GSE159115 | 14 |
| KIRC | GSE171306 | 2 |
| LIHC | GSE166635 | 2 |
| NF | GSE163028 | 3 |
| NHL | GSE147944 | 7 |
| NPC | GSE150430 | 16 |

**Table 1, Single-cell transcriptomic sample information (Continued)**

| **Cohort** | **Data set** | **Sample size** |
| --- | --- | --- |
| NSCLC | GSE143423 | 4 |
|  | GSE146100 | 3 |
| OS | GSE162454 | 6 |
| OV | GSE147082 | 6 |
|  | GSE154600 | 5 |
| PAAD | GSE111672 | 23 |
|  | GSE162708 | 5 |
| PPB | GSE163678 | 1 |
| PRAD | GSE141445 | 1 |
|  | GSE172301 | 13 |
| SCC | GSE144236 | 29 |
|  | GSE145328 | 11 |
| SKCM | GSE115978 | 7186 |
| SS | GSE131309 | 4 |
| STAD | GSE134520 | 13 |
|  | GSE167297 | 14 |
| THCA | GSE148673 | 13 |
| UVM | GSE160883 | 7 |

**Table 2, Spatial transcriptomic sample information**

| **Cohort** | **Data set** | **Spots number** |
| --- | --- | --- |
| CRC | 10× genomics | 3138 |
| PRAD | 10× genomics | 4317 |
| BRCA | GSE243275 | 4992 |
| EC | GSE225690 | 1091 |
| GBM | GSE235315 | 4764 |
| PAAD | GSE194329 | 3947 |

**Table 3, qRT-PCR primer sequences**

| **Gene name** | **Upstream Primers (5’ to 3’)** | **Downstream Primers (5’ to 3’)** |
| --- | --- | --- |
| 18S | GCAGAATCCACGCCAGTACAAGAT | TCTTCTTCAGTCGCTCCAGGTCTT |
| Vimentin | GACGCCATCAACACCGAGTT | GACGCCATCAACACCGAGTT |
| MMP-9 | GAGTTGAACCAGGTGGACCAAGTG | CTCCTCCCTTTCCTCCAGAACAGAA |
| Snail | TCGGAAGCCTAACTACAGCGA | AGATGAGCATTGGCAGCGAG |
| Slug | TCAAGGACACATTAGAACTCACACGG | CTACACAGCAGCCAGATTCCTCATG |
| Oct-4 | AGGATGTGGTCCGAGTGTGGT | CGAGGAGTACAGTGCAGTGAAGTG |
| Nanog | GCCGAAGAATAGCAATGGTGTGAC | GCTCCAGGTTGAATTGTTCCAGGT |
| RANBP1 | CATCCGCTTCCTGAATGCTGAGAAT | CATCCTCCTTGGTCTCCTCCTTCA |
| MAD2L1 | CAGCTGCTTAGACGCTGGATT | GTAGAAATACGGCTGCACCGA |
